# Chronic High-Fat Diet Does Not Alter Overall Cancer Incidence in *Trp53*^*R270H/+*^ Mice

**DOI:** 10.1158/2767-9764.CRC-25-0280

**Published:** 2026-06-08

**Authors:** Ilaria Panzeri, Zachary B. Madaj, Luca Fagnocchi, Stefanos Apostle, Megan Tompkins, Kristin Gallik, Eric H. Ma, Galen Hostetter, Russell G. Jones, John Andrew Pospisilik

**Affiliations:** 1 Department of Epigenetics, Van Andel Institute, Grand Rapids, Michigan.; 2 Bioinformatics and Biostatistics Core, Van Andel Institute, Grand Rapids, Michigan.; 3 Vivarium and Transgenics Core, Van Andel Institute, Grand Rapids, Michigan.; 4 Optical Imaging Core, Van Andel Institute, Grand Rapids, Michigan.; 5 Department of Metabolism and Nutritional Programming, Van Andel Institute, Grand Rapids, Michigan.; 6 Pathology and Biorepository Core, Van Andel Institute, Grand Rapids, Michigan.

## Abstract

Obesity is a complex chronic disease characterized by excessive adiposity and multiple comorbidities, including an elevated risk of several cancers. Despite long-standing epidemiologic links, the causal relationship between body mass index and reduced cancer survival remains controversial, underscoring the need to define how obesity-related factors—such as chronic high-fat diet (HFD) exposure—influence tumor development in the context of defined oncogenic lesions. Here, we report the effects of chronic HFD-induced obesity on cancer progression in *Trp53*^*R270H/+*^ mice, a model of human Li–Fraumeni syndrome characterized by spontaneous multicancer susceptibility. Surprisingly, despite rapid, sustained, and highly penetrant obesity, more than 1 year of HFD had no significant effect on overall survival, tumor burden, or tumor spectrum across more than 20 anatomic sites in *Trp53*-mutant mice. Crucially, we did identify a single, tissue-specific exception in the lung, with HFD-fed animals showing increased prevalence of lung tumors that correlated with unique, diet-specific changes in *Trp53* allelic and protein profiles. Notably, chronic HFD in this model triggered adipose-specific, not systemic, inflammation. These robust findings, replicated independently in two cohorts totaling 359 mice, suggest that *Trp53*^*R270H*^-driven multicancer syndrome is largely refractory to HFD-driven acceleration. Our results provide evidence that chronic diet-induced obesity does not universally enhance tumorigenesis, highlighting the complexity, tissue specificity, and context-dependent nature of obesity–cancer interactions.

**Significance:**

Obesity is widely linked to cancer risk, yet causality remains debated. Using large, longitudinal cohorts of Li–Fraumeni *Trp53*^*R270H/+*^ mice, this study shows that chronic HFD-induced obesity does not broadly accelerate tumor burden or shorten survival, revealing strong tissue specificity, with lung cancer as a notable exception.

## Introduction

Obesity is a chronic metabolic condition characterized by elevated body weight, adiposity, and numerous comorbidities, including cardiovascular disease, diabetes, and autoimmunity. Obesity (and more moderate “overweight”) affects ∼1.3 billion individuals globally, and the direct economic costs in the United States alone are estimated at $173 billion per year (1). In addition to cardiometabolic complications, obesity associates with increased incidence and severity of multiple cancers, including at least 13 distinct anatomic sites (2–5). Obesity has also been associated with altered treatment efficacy and toxicity (6, 7). Furthermore, obesity correlates with higher recurrence rates and poorer cancer prognoses (8), findings that have led to the inclusion of weight management in clinical guidelines for cancer survivors (9–11).

Although numerous mechanisms have been proposed to explain how obesity drives cancer (12–16), recent literature has questioned the causal association between body mass index (BMI) and reduced cancer survival (8, 17–26). This raises several important unanswered questions about the effects of obesity on cancer development and progression: *how* obesity influences tumorigenesis, *which* cancer types are susceptible to obesity-dependent regulation, and *when* during tumorigenesis is such regulation most relevant. This lack of understanding derives from methodologic issues, as well as questions of reverse causality, detection and selection biases, and the overall breadth of cancers observed in the clinic (27, 28).

Dietary composition and genetic susceptibility can profoundly influence obesity phenotypes in rodent models, limiting reproducibility (29). Obesogenic diets vary significantly in macronutrient composition, fat sources (e.g., saturated animal fats vs. unsaturated plant-derived fats), percentage of dietary fat, carbohydrate and protein content, as well as caloric density, all of which can differentially modulate metabolic, endocrine, inflammatory, and microbiome responses in rodents, ultimately influencing the outcomes of diet-induced obesity and associated pathologies (30–36). For example, diets rich in saturated fatty acids have been shown to drive stronger proinflammatory and insulin-resistant phenotypes than those enriched in unsaturated fatty acids, leading to distinct downstream effects on metabolic dysfunction and disease susceptibility (37–41). Similarly, variation in carbohydrate sources (simple sugars vs. complex carbohydrates) influences metabolic disease risk, inflammation, and tumorigenesis (42–46). Additionally, differences in dietary fiber content and micronutrient availability (e.g., vitamins, antioxidants, and trace minerals) also influence systemic inflammation, oxidative stress, and metabolic disease phenotypes (47–50). Genetic background further modulates susceptibility to obesity and its complications; common laboratory rodent strains (e.g., C57BL/6, BALB/c, FVB, and DBA) exhibit markedly different propensities toward diet-induced obesity, insulin resistance, and tumor susceptibility, emphasizing the necessity of careful selection and rigorous characterization of experimental models in obesity-cancer studies (51–63).

High-fat diet (HFD) feeding is the most commonly used murine model of obesity (64, 65). The utility of the model is underpinned by human epidemiologic studies that associate increased dietary fat intake with obesity incidence (66–68). Elevated dietary fat intake in humans and mice increases adiposity and associated hyperglycemia, inflammation, hypertension, plasma lipids, insulin resistance, and reduced β-cell function (69). Over time, this collectively leads to “metabolic syndrome” characterized by elevated adiposity, cardiovascular disease, hepatosteatosis, type 2 diabetes, and increased mortality (70, 71). Importantly, HFD treatment is just one of many murine models of human obesity (and modern Western diet composition). Just as human obesity is increasingly acknowledged as a heterogeneous set of disorders (72, 73) with numerous distinct genetic, epigenetic, and environmental drivers, HFD treatment only models a subset of the many recognized drivers of obesity. HFD has been shown to exacerbate a number of cancer models in mice, including colorectal cancer (74), hepatocellular carcinoma (75), pancreatic ductal adenocarcinoma (76), breast cancer (77), prostate cancer (78), and skin carcinogenesis models (79).

This study tested the interaction between chronic high-fat feeding and a *Trp53*-driven model of spontaneous murine multicancer syndrome (MCS), the *Trp53*^*R270H/+*^ mouse. *TP53* is one of the most commonly mutated tumor-suppressor genes in human cancers (80), and loss-of-function or dominant-negative mutations cause Li–Fraumeni syndrome (LFS; refs. 81–83). LFS is an autosomal dominant disorder characterized by a marked susceptibility to diverse p53-dependent cancers. *Trp53*^*R270H/+*^ mice (the equivalent of the human Li–Fraumeni hotspot mutation R273H) faithfully recapitulate many aspects of the human MCS disorder, exhibiting a broad spectrum of tumors, including a variety of carcinomas, soft tissue and bone sarcomas, leukemia, and even glioblastoma (the most common brain cancer in patients with LFS). *Trp53*^*R270H/+*^ mice exhibit a mean lifespan survival of approximately 1 year (83).

Here, we report a comprehensive longitudinal analysis of two large cohorts of *Trp53*^*R270H/+*^ animals challenged life-long with HFD or control chow diet (CD) from early adulthood. Surprisingly, despite robust and sustained induction of obesity and associated metabolic remodeling, HFD did not alter overall survival, tumor burden, or tumor spectrum in *Trp53*^*R270H/+*^ mice. The only tissue exhibiting a diet-dependent difference was the lung, where HFD animals also showed evidence of altered *Trp53* allelic and protein profiles. Together, these data highlight an important example in which chronic obesity does not accelerate or aggravate cancer etiology across many tumor types.

## Materials and Methods

### Origin and maintenance of mice

This research complies with ethical regulations and protocols approved by Institutional Animal Care and Use Committee (Van Andel Institute; protocols 19-0026, 22-09-036, 18-10-028, and 21-08-023). *B6.129S4-Trp53<tm3.1Tyj>/J* (*Trp53*^*R270H/+*^) animals were originally generated in The Jackson Laboratory (83) and purchased from The Jackson Laboratory (stock #008182, RRID: IMSR_JAX:008182). Mice were housed in the VAI Vivarium Core (RRID: SCR_023211) and Transgenic Core (RRID: SCR_022914); they were backcrossed for more than 10 generations and maintained in-house by breeding with wild-type (WT) siblings and periodic background refreshment using WTs from JAX (RRID: IMSR_JAX:000664). 392 F1 hybrids were generated by crossing 8-week-old *FVB.J* males (RRID: IMSR_JAX:001800) with two 8-week-old *B6.Trp53*^*R270H/+*^ females, which were separated after plug checking the next morning. Mating animals were randomly selected. All animals were fed breeder chow (LabDiet, 5021 cat. #0006540) *ad libitum* upon weaning and then randomly assigned to breeder chow (CD) or high-fat diet (HFD, Research Diets, D12492i) at 8 weeks of age. Breeder chow was selected as control diet to ensure consistency with the animals’ dietary history. We note that unlike the purified-ingredient HFD, the breeder chow contains plant-derived ingredients, including soy, and thus higher phytoestrogen and antioxidant content. These compositional differences are detailed in Supplementary Table S1. Mice were housed in individually ventilated cages (Tecniplast, Sealsafe Plus GM500 in DGM Racks) at a density of maximum five animals per cage. Each cage was enriched with Enviro-dri (The Andersons, Crink-l’Nest) and cardboard dome homes (Shepherd, Shepherd Shack Dome). Whenever possible, same-sex siblings and same-sex animals from different litters were combined (∼20 days of age). Animals were kept on a 12-hour light/dark cycle at an average ambient temperature of 23°C and 35% humidity.

Body composition data were collected from 359 animals, including 173 males (74 WT and 99 *Trp53*^*R270H/+*^) and 186 females (82 WT and 104 *Trp53*^*R270H/+*^). CD (LabDiet, 5021 cat. #0006540) or HFD (Research Diets, D12492i) was randomly assigned at 8 weeks of age, as follows: 24 WT males under CD, 29 WT males under HFD, 38 *Trp53*^*R270H/+*^ males under CD, 56 *Trp53*^*R270H/+*^ males under HFD, 30 WT females under CD, 40 WT females under HFD, 41 *Trp53*^*R270H/+*^ females under CD, and 60 *Trp53*^*R270H/+*^ females under HFD. At 4, 8, 16, 32, 40, 50, 60, and 70 weeks of age (or at euthanasia), mice were weighed and scanned via EchoMRI for fat and lean mass composition in the morning (EchoMRI, EchoMRI-100H).

Tumor analysis was conducted on 182 animals: 85 males (7 WT-CD, 10 WT-HFD, 26 *Trp53*^*R270H/+*^-CD, and 42 *Trp53*^*R270H/+*^-HFD) and 97 females (8 WT-CD, 13 WT-HFD, 30 *Trp53*^*R270H/+*^-CD, and 46 *Trp53*^*R270H/+*^-HFD). Twenty-five animals were found deceased (9 *Trp53*^*R270H/+*^ HFD, 5 *Trp53*^*R270H/+*^ CD, and 3 WT HFD), and tumor evaluation was not possible in 17 cases due to postmortem degradation or perinatal death. We performed tumor analysis blinded for genotype and phenotype, temporally collecting mice according to the timing of health reports. We specify in the text every time we are only referring to one of the sexes. All cohort data are provided in Supplementary Table S2.

### Statistics and reproducibility

Power analysis was performed by the Van Andel Institute (VAI) Bioinformatics and Biostatistics Core (RRID: SCR_024762) using the pwr R package for Power Analysis (R v.3.5.2) to determine sample size. In particular, a two-sample test of proportions was run based on an estimated effect size calculated on published data (83) using a firth logistic regression. Power was set to 80%, α = 0.05, and assuming each group having equal sample sizes, considering two treatments (CD and HFD) and five different types of cancer evaluated (germ cell tumors, carcinoma, sarcoma, lymphoma, and leukemia).

Due to COVID-related reductions, 165 animals were randomly excluded for tumor analysis. Additionally, seven mice died after birth, precluding further analysis. Ten mice were found dead and too stiff to harvest. The final cohort included all animals from litters of 5 to 12 pups.

Experiments were randomized, and investigators were blinded to group allocation and outcome assessment wherever possible.

Weight and mass trajectory data were analyzed using generalized additive models in R v4.5.0 with the mgcv package (84). For each outcome, two models were fit: one including splines-by-diet and one with only a spline for age. Both models included a random effect spline to account for repeated measures on each mouse. A likelihood ratio test, implemented via the lmtest package (85), was used to compare models; a significantly better fit of the splines-by-diet model indicated evidence for diet-specific trajectories.

To test for diet differences in the incidence of tumor location and/or tumor type, Poisson regressions with a log-offset for time until euthanasia were used. To account for multiple testing across incidence rates and prioritize meaningfully different results, second-generation *P* values were calculated using the sgpv package, with a null interval of ±10%, similar to the approach used by Blume and colleagues (86, 87).

Jensen–Shannon divergence (JSD), a symmetric measure of dissimilarity between probability distributions, was used to quantify differences in the distributions of cancer type and tissue of origin between HFD and CD. JSD ranges from 0 to 1, with 0 indicating identical distributions and higher values indicating greater divergence. JSD was computed using base-2 logarithms (88).

### Genotyping

Ear punch biopsies were collected at 10 days and digested in 20 μL genomic DNA lysis buffer (100 mmol/L Tris-HCl pH 8.5, 5 mmol/L EDTA, 0.2% SDS, and 100 mmol/L NaCl) with 20 mg proteinase K (Thermo Fisher Scientific, EO0491). The thermal cycling protocol used was 55°C for 16 hours, 95°C for 10 minutes, and a 4°C hold (lid at 105°C). Nuclease-free water (Invitrogen, AM9938) was added to each lysate for a final volume of 180 μL. PCR reactions for *Trp53* allele used 1 μL diluted biopsy lysate in a 19 μL master mix (1× DreamTaq Buffer, 0.2 mmol/L dNTPs, 0.1 μmol/L primer forward and reverse mix, and 2 U DreamTaq DNA Polymerase in nuclease-free water; Thermo Fisher Scientific, EP0703). PCR primer and thermal cycling conditions are detailed in Supplementary Tables S3 and S4. Twenty microliters of each PCR product were digested with 0.5 μL MslI (for *Trp53*^*R270H*/+^; New England BioLabs, R0571L) in 3 μL CutSmart Buffer and nuclease-free water, in a final reaction volume of 30 μL. Restriction conditions are detailed in Supplementary Table S5. Digestion products (∼500 bp WT *Trp53*, ∼200 + ∼300 bp *Trp53*^*R270H/+*^) were visualized on a 3% agarose gel Thermo (Fisher Scientific, BP160-500) in 1× TAE, with GelRed as intercalating dye (Biotium, 41003).

### Health monitoring

VAI Vivarium Core staff monitored mice 2 to 3 times per week for health, well-being, and abnormal mass/tumor presence. Mice were euthanized if they exhibited >20% weight loss, tumors ∼15% of body weight (this maximal tumor size was never exceeded), tumor ulcerations, tumor discharge or hemorrhage, mobility issues, reduced appetite or hydration, limited defecation or urination, abnormal gait or posture, labored breathing, lack of movement, or hypothermia. Mice with reported health concerns or those reaching the 70-week study endpoint were euthanized via CO_2_ asphyxiation and cervical dislocation.

### Tissue harvesting

Tissues were dissected and fixed in 10% NBF solution (3.7%–4% formaldehyde 37%–40%, 0.03 mol/L NaH_2_PO_4_, and 0.05 mol/L Na_2_HPO_4_ in distilled water with a final pH of 7.2 ± 0.5): epididymal white adipose tissue (eWAT); uterus or preputial glands, seminal vesicles, and testis; bladder; pancreas; spleen; intestine; stomach; mesenteric fat; liver; kidneys; heart; lungs; thymus; brain; breast (ninth); and hindlimb muscles and bones. We also recovered spine, ribs, skull, skin, and any other mass if abnormal. Fixative volume was 15 to 20 times the tissue volume. Specimens >2.5 mm thick were cut to ensure proper fixation. Most tissues were fixed for 40 hours, whereas fat-rich tissues (eWAT, mesenteric fat, and uterus) were fixed for 72 hours. Bones and spines were fixed for 1 week, followed by 1-week decalcification in 14% EDTA (14% free-acid EDTA at pH 7.2, adjusted with NH_4_OH). After incubation, all tissues were moved to 70% ethanol. Data collection was blinded.

### Tissue preparation for histology

All tissues were paraffin-embedded by the VAI Pathology and Biorepository Core (RRID: SCR_022912). Dehydration and clearing were automated with a Tissue-Tek VIP 5 (Sakura) using the following protocol: 60 minutes in 70% ethanol; 60 minutes in 80% ethanol; 2 × 60 minutes in 95% ethanol; 3 × 60 minutes in 100% ethanol; 2 × 30 minutes in xylene; and 75 minutes in paraffin. Embedding was performed with a Leica EG1150. Three 5-μm sections, spaced 150 μm apart, were cut from each tissue for hematoxylin and eosin (H&E) staining using a Leica rotary microtome. The remaining tissue was stored as a paraffin block. H&E staining was performed using a Tissue-Tek Prisma Plus Automated Slide Stainer (Sakura) and Prisma H&E Staining Kit #1.

### Pathology evaluation

Standard 5-μm H&E-stained sections were assessed for tumors and dysplastic lesions by a board-certified pathologist at the VAI Pathology and Biorepository Core. Most samples were provided blindly. Tumors were classified as malignant or benign, with all malignant tumors being primary. Metastatic or secondary tumors were identified based on primary tumor characteristics and immunohistochemical validation but were not analyzed in this study. Tumors were categorized into carcinomas, germ cell tumors, leukemias, lymphomas, and sarcomas, with detailed classification by tissue of origin.

### Tissue preparation for DNA extraction

Tumor areas and contralateral healthy tissues were macrodissected from formalin-fixed, paraffin-embedded (FFPE) tissues by manually using a razor blade. The mounted sections were cut at 10 μm and dried in an oven at 60°C for 20 minutes. All slides were dissected within 24 hours of sectioning. Dissected areas were stored at 4°C in DNA/RNA Shield (Zymo, R1100) until further processing.

### DNA extraction from tissues

DNA from FFPE tumor and healthy tissue sections was extracted using the QIAamp DNA FFPE Advanced Kit (QIAGEN, 56604) with slight modifications. DNA/RNA Shield (Zymo, R1100) was removed by pipetting to leave the tissue sections as dry as possible. Subsequent protocol steps were followed according to the kit manual. Elution was performed in 50 μL of Buffer ATE and then reapplied to the spin column for an additional 3 minutes at room temperature to increase DNA yield.

### Digital droplet PCR

Prior to digital droplet PCR (ddPCR), gDNA samples were digested using HindIIIHF (New England Biolabs). Thirty nanograms of digested material was input into ddPCR reactions prepared using ddPCR Supermix for Probes (no dUTP; Bio-Rad) and Custom ddPCR FAM/HEX Assays (Bio-Rad). The assay was run using the primers and probes listed in Supplementary Table S6. Droplets were generated and read utilizing the QX200 AutoDG Droplet Digital PCR System (Bio-Rad). Fluorescence droplet data were analyzed via QX Manager Standard Edition 2.2 (Bio-Rad). These experiments were performed in the VAI Genomics Core (RRID: SCR_022913).

### Immunofluorescence staining

Samples were randomly selected based on tumor type, genotype, and diet. Paraffin sections (5 μm) were deparaffinized and subjected to antigen retrieval using DAKO EnVision FLEX High pH antigen retrieval buffer for 20 minutes at 97°C by the VAI Pathology and Biorepository Core. Slides were blocked with 2% FBS for 1 hour and then incubated overnight at 4°C with 1:100 anti-CD45 (Abcam, ab10558), 1:600 S100 (Invitrogen, MA5-32625), 1:500 Ki67 (Invitrogen, 740008T), and 1:200 P53 antibodies (Leica, LCL-L-p53-CM5p) and, depending on the experiment, diluted in DAKO EnVision FLEX antibody diluent. After washing 3× with 1× PBS for 5 minutes, slides were stained with Rb-647 secondary antibody (Invitrogen, A32733) at 1:500 for 2 hours at room temperature, followed by three washes with 1× PBS for 5 minutes each. DAPI (Invitrogen, D21490) was applied for 10 minutes at room temperature, and slides were washed 3× in DI water for 5 minutes each before coverslipping with ProLong Gold mounting medium (Invitrogen, P36930).

### Immunofluorescence acquisition

Whole-tissue images were collected in a single plane on a Zeiss Axioscan 7 slide scanner using ZEN blue (version 3.7) by the VAI Imaging Core (RRID: SCR_021968). DAPI- and AF647-stained samples were excited by a Colibri 7 LED light source at 385 and 630 nm. Emission was collected through a Zeiss Plan-Apochromat 20x, 0.8-NA air objective and detected with a Zeiss Axiocam 712 camera. Resulting 14-bit images, scaled to 0.1725 × 0.1725 μm per pixel, were compressed by JpgXr at 85%. Data collection and analysis were conducted blind to the experimental conditions.

### Immunofluorescence image analysis

All custom models, classifiers, and scripts used for the following image analysis pipelines are publicly available in a GitHub repository: https://github.com/vaioic/Panzeri_Image_Analysis_Methods.

Large batch analyses for completed pipelines were run on VAI’s HPC (Linux) via Virtual Desktop in OnDemand either using a GPU node with 4 Nvidia L40s, 768 GB RAM, and 196 cores or a CPU node with 40 cores and 256 GB RAM (GPU node use indicated below). Pipeline development and smaller batch runs were carried out on a Silicon Mechanics workstation running Windows 11 with an Intel Xeon W-3223 CPU, 512 GB RAM, NVIDIA RTX A4000 GPU, and 16TB Accelsior 8M2 SSD.

All image analyses described in the following sections were carried out in QuPath (v5.1; ref. 89). QuPath projects were separated by experiment. Original czi image files were imported into QuPath and read using Bio-Formats. Annotation outlines of each tissue were generated using the QuPath SAM extension (v0.7.0) with model vit_h (huge), default weights, and rectangle draw prompt with GPU acceleration (bioRxiv 2023.06.13.544786; arXiv 2312.09579; https://github.com/ksugar/qupath-extension-sam). Manual outline correction was applied as needed to remove large holes and bright autofluorescence regions caused by debris. Specific analyses are detailed in the following sections. Any additional parameters available for the plugins or tools used that are not specifically mentioned were left at their default values.

#### CD45 – eWAT

To measure the area of CD45 staining, a training image composed of regions of interest (ROI) from all tissues representing the variability in CD45 staining was created with QuPath’s Create Training Image built-in tool. A global threshold value for the CD45 channel was calculated with the Huang threshold algorithm applied to the training image with 2 × 2 pixel binning to ease computational load. The calculated value was used in a pixel classifier created with QuPath’s Create Thresholder set to 0.345 μm/pixel resolution (equivalent to the binning used previously). This classifier was used to measure the CD45 area in each tissue and generate a detection object outlining the signal. A publicly available script (https://github.com/yau-lim/QuPath-Auto-Threshold) was used for calculating the threshold with the Huang algorithm. The area of the detected CD45 signal and the area of the tissue were exported as a CSV file to calculate the ratio of tissue occupied by CD45.

#### CD45 – lung tissues

The Moments threshold algorithm was applied—using the same threshold script from the CD45 – adipose tissue section—to the CD45 channel of each image with 4 × 4 pixel binning to measure the area of CD45 staining and create a detection object outlining the positive pixels. The area of the detected CD45 signal and the area of the tissue were exported as a CSV file to calculate the ratio of tissue occupied by CD45.

#### CD45 – uterus, seminal vesicles, and prostate

To measure the tissue area occupied by CD45 staining, a pixel classifier was trained for the uterus, seminal vesicles, and prostate tissues independently (i.e., one model for each tissue) using QuPath’s built-in Train Pixel Classifier set to a down sampled resolution of 0.69 μm/pixel. A pixel classifier was used for these samples instead of a threshold algorithm to accommodate for the unique intersample and intrasample variability in tissue autofluorescence. For each pixel classifier trained, a Random Trees (RTrees) model was used with the following parameters: maximum depth = 5 and maximum number of trees = 100. The features used for training were Gaussian, Laplacian of Gaussian, weighted standard deviation (SD), structure tensor eigenvalue maximum, and structure tensor eigenvalue minimum each at scales of 0.5, 1, 2, and 4 for both the DAPI and CD45 channels. Local mean subtraction with a radius of 20 was additionally used to aid normalization within and across tissues. All CD45-stained tissues were used for training due to smaller sample size and variability mentioned above. Closed polygon annotations were used to annotate examples of CD45 staining and background. Sparse labeling was used to avoid overtraining the model. Each model achieved an accuracy of ∼80% or greater. A custom script was then used to apply the pixel classifier, measure the area of detected pixels, and create detections from the classifier results. The area of the detected CD45 signal and the area of the tissue were exported as a CSV file to calculate the ratio of tissue occupied by CD45.

#### p53 – lung, prostate, seminal vesicles, and uterus

The same areas identified for ddPCR as tumor and contralateral normal in each tissue type were used for p53 protein expression quantification. Two training images were generated with QuPath’s Create Training Image built-in tool—one for the prostate, seminal vesicles, and uterine tissues, and the other for the lung tissues due to different imaging parameters. These training images were used to determine cell detection parameters and train two separate object classifiers. Cell detections were generated in the tissue ROIs using CellPose3’s (v0.3.1.1.1) cyto3 model via the CellPose QuPath extension (v0.9.6) with GPU acceleration (https://github.com/BIOP/qupath-extension-cellpose; refs. 90, 91). The following customized parameters were used: pixel size = 0.1725 and channels = 0, 1. A specified temporary directory was used to allow parallel jobs from the same project to run. The following customized parameters were used: median filter = 2, tile size = 4,096, diameter = 30, measure shape and intensity enabled, and no cell expansion used. The additional parameter addParameter (“gpu_device”, gpu index) was used for parallel processing with multi-GPU support, with each batch sent to a different GPU on a multi-GPU node at VAI’s HPC (4 Nvidia L40s 48 GB VRAM, 768 GB memory, 196 cores). To remove false-positive nuclei detections, an object classifier was trained using QuPath’s built-in Train Object Classifier with an RTrees model and all detection measurements available. Point annotations were used to label examples of true and false nuclei in the training images. All false nuclei detections labeled by the object classifier were removed from further analysis. Detection fluorescence measurements were then exported as a CSV file for further processing and reported as tumor over normal p53 intensities.

#### S100 and Ki67 – eWAT

To calculate the ratio of proliferative and nonproliferative adipocyte tissues, samples dual-stained with S100 and Ki67 were analyzed. QuPath’s built-in SLIC superpixel segmentation tool was used to generate detections in each tissue by grouping similar pixels using the following parameters: Gaussian sigma = 1; superpixel spacing = 20 (92). The Calculate Intensity Features tool in QuPath was then run to measure the following fluorescence intensity measurements for all three channels (DAPI, s100, and ki67) at full resolution (0.172 μm/pixel) in all SLIC superpixel detections: mean, SD, minimum, maximum, median, and Haralick features (minimum: 0; maximum: 10,000). Because adipocyte tissue contains many holes from the large fat reservoirs, SLIC superpixel detections occupying these areas were removed by filtering for a Ki67 SD value less than or equal to 100. An object classifier was then trained using QuPath’s built-in Train Object Classifier to classify SLIC superpixel detections as proliferative or nonproliferative. An artificial neural network model was used with the first layer containing 52 nodes (equal to the number of features used for training) and a second layer of two nodes (equal to the number of classes being trained). Features were normalized using mean and variance. All tissues were used for training due to smaller sample size and variability between samples. Point annotations were used to annotate examples of proliferative (dual-positive and thicker extracellular matrix) and nonproliferative (not dual-positive and thinner extracellular matrix) SLIC superpixel detections, and sparse labeling was used to avoid overtraining the model. For each tissue, all SLIC superpixel detections classified as proliferative were converted into a merged annotation to easily measure the total area of proliferative regions. The areas of the whole tissue and proliferative regions were exported as a CSV file for further analysis and reported as the ratio of proliferative over total tissue area.

### Immunophenotyping

Blood was collected from animals at 40 weeks of age by tail nicking and placed on ice immediately after collection. Tubes were centrifuged at 500 *g* for 5 minutes in a precooled centrifuge (4°C), and the supernatant was discarded. Red blood cell lysis was performed for 5 minutes at room temperature in 1× Red Blood Cell Lysis Buffer (Invitrogen, 00-4300-54). Lysate was centrifuged at 500 *g* for 5 minutes at 4°C. The supernatant was then discarded, and the pellet was resuspended in 200 μL FACS buffer (1× PBS, 2% FBS, and 0.05% w/v NaN_3_) and plated in a 96-well round-bottom plate. A centrifugation step was repeated (500 *g* for 5 minutes at 4°C), the supernatant discarded, and single-cell suspensions surface-stained with a 1:300 dilution of fluorescently conjugated antibodies against murine CD19-FITC (eBioscience, 11-0193-85), NK1.1-PE (BioLegend, 108708), CD44-PE-Cy7 (eBioscience, 25-0441-82), CD4-APC (eBioscience, 17-0042-82), CD3-eFluor450 (eBioscience, 48-0032-80), and CD8-BV510 (BD Bioscences, 563068). Cell viability was assessed using a 1:1,000 dilution of eFluor-780 Fixable Viability Dye (eBioscience, 65-0865-18) following the manufacturer’s protocol. Staining was performed for 1 hour at 4°C in the dark. Flow cytometry was performed on Cytek Aurora Spectral Flow Cytometry at the VAI Flow Cytometry Core (RRID: SCR_022685). Analysis was performed with FlowJo software (Tree Star).

### Plasma collection

Blood was collected from animals at 8, 16, and 40 weeks of age by tail nicking directly harvesting in EDTA-coated Microvettes (Sarstedt, 16.444.100) to reduce hemolysis, which were placed on ice immediately after collection. Plasma was separated by centrifugation within 1 hour of collection at 4,000 *g* for 10 minutes in a precooled centrifuge (4°C). The supernatant was collected in precooled 1.5-mL receiving tubes and snap-frozen in liquid nitrogen. Plasma was then placed at −80°C for long-term storage.

### LEGENDplex multianalyte flow assay

Frozen plasma aliquots were thawed on ice, mixed well, and centrifuged to remove particulates at 4,000 *g* for 10 minutes at 4°C before use. Samples were then run in technical duplicates using a 10-fold dilution following the manufacturer’s instruction for a custom mouse panel LEGENDplex Multi-Analyte Flow Assay using a V-bottom plate targeting CCL2 (MCP-1), CXCL10 (IP10), VEGF, IFNγ, IL11, IL18, IL1β, IL22, IL6, MCSF, TNFα, and TGF1β, with minor modifications. Reduced acceleration and brake were used every time the plate was centrifuged, and flow acquisition was set to 2,000 beads per analyte instead of the 300 recommended in the manual to achieve better representation of each analyte. Samples were acquired using a Beckman Coulter CytoFLEX S. Data analysis was performed using BioLegend’s LEGENDplex data analysis software. IL10 was included in the panel but consistently detected below background and therefore excluded from the reported data.

### Lipid extraction

Lipids were extracted by the VAI Mass Spectrometry Core (RRID: SCR_024903). Briefly, they were separated by mixing 40 μL of plasma in 1 mL of chloroform:methanol:water (2:2:1.8 v/v; refs. 93, 94), incubating on wet ice for 1 hour, and centrifugation at 14,000 *g* for 10 minutes to induce phase separation. The upper aqueous and bottom organic layers (495 and 100 μL, respectively) were collected into separate tubes and dried in a speedvac. The organic layer was resuspended 200 μL of 50:50 (isopropanol:acetonitrile, v/v) for lipidomics analysis.

### Lipidomics spectrometry

Lipidomics samples were analyzed with a Thermo Vanquish dual liquid chromatography system utilizing two alternating methods, referred to as chromatography 1 and chromatography 2, coupled to an Orbitrap ID-X (Thermo Fisher Scientific) using an H-ESI (heated electrospray ionization) source in positive and negative modes, respectively. Two microliters of each standard and/or sample were injected, column temperatures were kept at 50°C, and flow rate was held at 0.4 mL/minute. For both chromatography 1 and 2, mobile phase A consisted of 60% liquid chromatography/mass spectrometry (LC/MS)-grade acetonitrile (A955, Thermo Fisher Scientific), 40% LC/MS-grade water (W6, Thermo Fisher Scientific), 0.1% LC/MS-grade formic acid (A117, Thermo Fisher Scientific), and 10 mmol/L ammonium formate (70221, Thermo Fisher Scientific), and mobile phase B consisted of 90% LC/MS-grade isopropanol (A461, Thermo Fisher Scientific), 8% LC/MS-grade acetonitrile, 2% LC/MS-grade water, 0.1% LC/MS-grade formic acid, and 10 mmol/L ammonium formate. Chromatography 1 used a 30-minute reversed-phase chromatography Accucore C30 column (2.6 μm, 2.1 mm × 150 mm, 27826-152130, Thermo Fisher Scientific) combined with an Accucore C30 guard column (2.6 μm, 2.1 mm × 10 mm, 27826-012105, Thermo Fisher Scientific), and the gradient was as follows: 0 to 1 minutes held at 25% B, 1 to 3 minutes from 25% B to 40% B, 3 to 19 minutes from 40% B to 75% B, 19 to 20.5 minutes 75% B to 90% B, 20.5 to 28 minutes from 90% B to 95% B, 28 to 28.1 minutes from 95% B to 100% B, and 28.1 to 30 minutes held at 100% B. A 30-minute wash gradient was run between every injection (in parallel with chromatography 2) to flush the column and reequilibrate solvent conditions as follows: 0 to 2 minutes held at 100% B and 0.3 mL/minute, 2 to 2.1 minutes from 100% B to 25% B and held at 0.3 mL/minute, 2.1 to 4 minutes held at 25% B and ramp to 0.4 mL/minute, 4 to 6 held at 25% B and ramp to 0.6 mL/minute, 6 to 17 minutes held at 25% B and 0.6 mL/minute, 17 to 17.1 minutes held at 25% B and ramp to 0.4 mL/minute, and 17.1 to 30 minutes held at 25% B and 0.4 mL/minute. Chromatography 2 used a 30-minute reversed-phase chromatography Acquity UPLC CSH C18 column (1.7 μm, 2.1 mm × 100 mm, 186005297, Waters, Eschborn, Germany) combined with a VanGuard precolumn (1.7 μm, 2.1 mm × 5 mm, 186005303, Waters), and the gradient was as follows: 0 to 1 minutes held at 25% B, 1 to 3 minutes from 25% B to 40% B, 3 to 4 minutes from 40% B to 50% B, 4 to 16 minutes from 50% B to 65% B, 16 to 17 minutes from 65% B to 70% B, 17 to 25 minutes from 70% B to 75% B, 25 to 27 minutes from 75% B to 100% B, and 27 to 30 minutes held at 100% B. A 30-minute wash gradient was run between every injection (in parallel with chromatography 1) that used the same gradient as chromatography 1 wash gradient. For both methods mass spectrometer parameters were as follows: source voltage +3,250 V or −3,000 depending on method polarity, sheath gas 40, aux gas 10, sweep gas 1, ion transfer tube temperature 300°C, and vaporizer temperature 275°C. Full scan data were collected using the Orbitrap with a scan range of 200 to 1,700 m/z at a resolution of 500,000 and RF lens at 45%. Data-dependent MS2 fragmentation was induced in the Orbitrap using assisted higher-energy collisional dissociation collision energies at 15, 30, 45, 75, and 110% as well as with collision-induced dissociation (CID) at a collision energy of 35%. For both MS2 fragmentations, Orbitrap resolution was 15,000 and the isolation window was 1.5 m/z. A m/z 184 mass trigger, indicative of phosphatidylcholines, was used for CID fragmentation. Data-dependent MS3 fragmentation was induced in the ion trap with scan rate set at rapid using CID at a collision energy of 35%. MS3 scans were triggered by specific acyl chain losses for detailed analysis of monoglycerides, diglycerides, and triacylglycerides. Total cycle time was 2 seconds. Lipid identifications were assigned using LipidSearch (v5.0, Thermo Fisher Scientific).

### Lipidomics analysis

To determine which lipids were differentially abundant between the HFD and normal CD, semi-parametric differential abundance models were fit using SDAMS (87, 95) at each time point. This method models lipid detection (detected/not detected) and average abundance as a mixture, allowing *P* values to jointly account for both mean differences in abundance and group-specific lipids (i.e., lipids primarily present in one diet or time point but not others). The mixture-model approach made specification of an appropriate null interval difficult; therefore, multiple testing correction was performed using the Benjamini–Hochberg false discovery rate (FDR), and features with FDR <0.05 were considered significant. Of the 4,256 molecules tested, 569 could be confidently annotated. Molecules that were confidently annotated and differentially abundant were submitted to LIPEA (https://hyperlipea.org/faq), with the background universe set as the full 569 annotated compounds, to test for pathway enrichments. Of the remaining 3,687 lipids, approximate compounds were identified using LipidMaps (https://www.lipidmaps.org/bulk_search); these are denoted as “approximate annotation” throughout the article. The results were visualized as heatmaps using the ComplexHeatmap package (96). Lipidomics data can be found in MassIVE repository (ucsd.edu) with the dataset identifier MSV000101772.

## Results

### A cohort to study chronic effects of obesity on *Trp53*-dependent cancers

**Figure 1.**
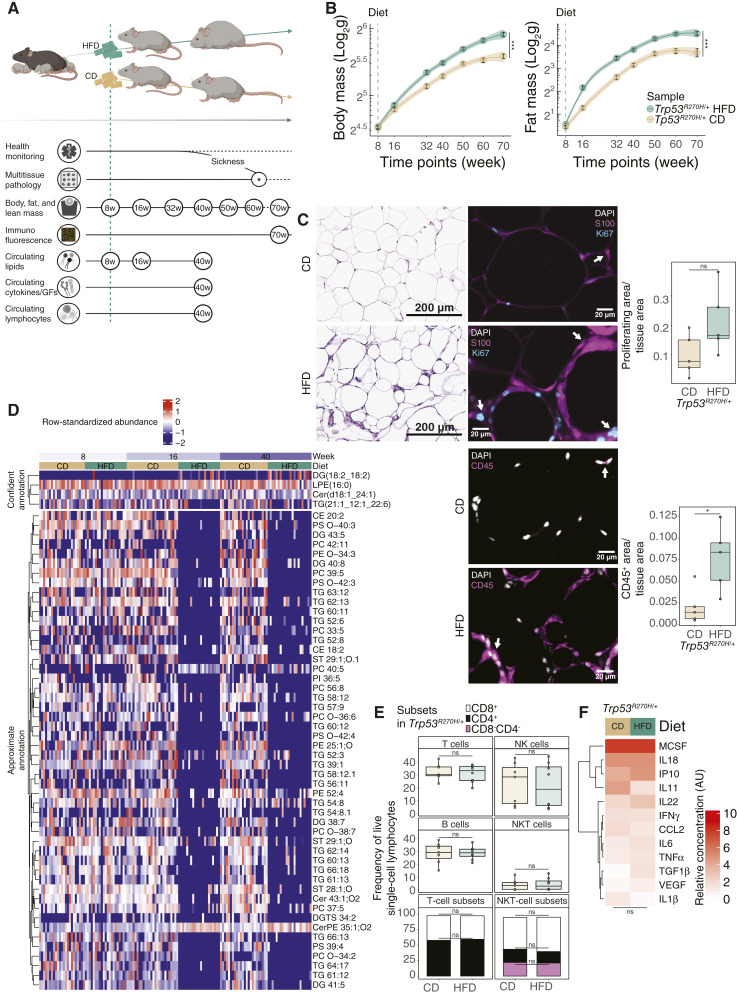
HFD induces metabolic remodeling and eWAT-restricted inflammation without systemic immune activation in *Trp53*^*R270H/+*^ mice. **A,** Schematic of the experimental plan. B6.*Trp53*^*R270H/+*^ females were mated with WT FVB.J males. F1 genotypes were fed CD or HFD starting at 8 weeks of age and screened for health issues and mass development. Tissues were harvested at sickness report. Histopathology determined the presence of tumors. Immunofluorescence analysis was conducted in adipose tissue at the endpoint. Body, fat, lean mass measurements, and circulating plasma for lipidomic analysis were collected at multiple time points. Circulating cytokines, growth factors (GF), and immunophenotyping were analyzed at 40 weeks of age. w, weeks. **B,** Scatter plots and smoothed conditional means (95% confidence interval, “loess” method) for body (left) and fat (right) mass in *Trp53*^*R270H/+*^ female and male mice (pooled data) under CD (beige) or HFD (green). Data are reported as log_2_ scale. *N* = 195 animals (101 female and 94 male mice). Significant differences were observed for CD vs. HFD body and fat mass trajectories with generalized additive models with random effects (likelihood ratio test): *P* = 3.577767e−16 for body mass and *P* = 1.266577e−15 for fat mass. **C,** Representative examples of H&E- (left), S100- and Ki67- (middle), and CD45-stained (right) eWAT from CD- (top) and HFD-fed (bottom) *Trp53*^*R270H/+*^ mice at 70 weeks of age (*N* = 1 male mouse), with relative quantifications (*N* = 5 CD-fed and *N* = 5 HFD-fed male mice). CD45- or S100-positive cells are shown in magenta, Ki67-positive cells in cyan, and DAPI-stained nuclei in white. Arrows indicate examples of positive signals. Scale bar, 100 μm for H&E and 20 μm for immunostaining. No significant differences (ns) were observed in the proliferating vs. nonproliferating area CD vs. HFD comparison with Wilcoxon rank-sum exact test: W = 4, *P* = 0.09524. Significant differences were observed in the CD45^+^ area CD vs. HFD comparison with Wilcoxon rank-sum exact test: W = 2, *P* = 0.03175. **D,** Heatmap of differentially abundant lipid species in circulating plasma from *Trp53*^*R270H/+*^ mice CD- or HFD-fed at 8 (before diet), 16, and 40 weeks of age. Each column represents an individual animal; rows correspond to lipid species grouped by annotation confidence. Row-standardized abundance values are shown (red = higher, blue = lower abundance). *N* = 63 animals (18 CD-fed females and 15 CD-fed males, 15 HFD-fed females and males). **E,** Immunophenotyping of circulating lymphocytes at 40 weeks. Quantification of T cells (CD19^−^CD3^+^), NK cells (CD19^−^CD3^−^NK1.1^+^), B cells (CD19^+^), and NKT cells (CD19^−^CD3^+^NK1.1^+^) in blood from CD and HFD *Trp53*^*R270H/+*^ animals, expressed as frequency of live single-cell lymphocytes. Bar plots show distribution of CD4^+^ and CD8^+^ T-cell and NKT-cell subsets. *N* = 15 animals (7 CD and 8 HFD-fed, pooled female and male data). No significant differences (ns) were observed for population CD vs. HFD comparison with Wilcoxon rank-sum test (normal approximation) followed by a Benjamini–Hochberg (BH) correction: *P*adj = 1 for all comparisons, W = 29.5 (B), 30.5 (NK), 24 (NKT), 28 (T and CD4^−^CD8^−^NKT), 21 (CD4^+^ T), 32.5 (CD8^+^ T), 33 (CD4^+^ NKT), and 25 (CD8^+^ NKT). **F,** Heatmap of circulating cytokine and growth factor levels in plasma at 40 weeks, measured by LEGENDplex assay. Rows indicate individual analytes, and columns CD- or HFD-fed *Trp53*^*R270H/+*^ mice. Color scale denotes relative concentrations (*N* = 30 animals, 13 CD-fed and 17 HFD-fed, pooled female and male data). No significant differences (ns) were observed between analytes comparison with Wilcoxon rank-sum exact test followed by a BH correction: *P* = 0.864 (IFNγ and TGF1β), 0.840 (IL1β, IL6, and TNFα), 0.900 (CCL2, IL11, IL18, IL22, IP10, MCSF, and VEGF). ***, *P* < 0.001; *, *P* < 0.05. [**A,** Created in BioRender. Panzeri, I. (2026) https://BioRender.com/zj95vt3.]

We set up two independent cohorts in series, totaling 359 animals, with equal representation of females and males. The cohorts included 203 *Trp53*^*R270H/+*^ animals and 156 WT littermates to control for potential developmental effects (97). All mice were fed a regular CD until 8 weeks of age, when they were randomly assigned to CD or HFD groups for the remainder of the experiment (the composition of these diets is detailed in Supplementary Table S1). We tracked animals from birth to the predetermined endpoint of 70 weeks of age (more than 1 year of HFD) regularly monitoring morphologic, growth and metabolic characteristics, as well as performing health checks for signs of cancer onset 2 to 3 times per week (Fig. 1A). At sacrifice, 182 animals were randomly selected for tumor analysis and underwent a standardized 21-organ dissection protocol in which tissues were isolated, processed for histology, and scored by a board-certified pathologist (all cohort data are provided in Supplementary Table S2).

Mice of both genotypes and sexes responded rapidly and appropriately to the HFD intervention with significant gains in overall fat and whole-body masses (Fig. 1B; Supplementary Fig. S1A–S1F). Notably, given previous reports of metabolic roles for WT p53 (98), we found no differences in relative body or fat mass deposition comparing *Trp53*^*R270H/+*^ and WT animals (Supplementary Fig. S1B and S1F).

Immunofluorescence analysis revealed a trend toward increased S100^+^/Ki67^+^ proliferative adipose stromal/lipoblast-like clusters in eWAT from HFD-fed animals and a significant rise in immune cell infiltration (increased CD45^+^ cell area in HFD- compared with CD-fed animals; Fig. 1C; Supplementary Fig. S1C). Such adipose tissue remodeling, characterized by expansion of proliferative stromal/progenitor populations and immune cell recruitment, is a hallmark of HFD-induced obesity and associates with progressive fibrosis and local chronic inflammation in eWAT (99, 100). These processes have been linked to systemic metabolic stress and the formation of protumorigenic or tumor-supportive niches in several preclinical cancer models, in which obese adipose depots contribute inflammatory and metabolic cues that enhance tumor initiation and progression (101, 102). Further supporting an effect of HFD, lipidomic profiling revealed broad alterations in circulating lipid species across multiple families in HFD-fed mice, including time- and group-specific effects, with the most pronounced alterations observed in HFD-fed animals at 16 and 40 weeks (Fig. 1D).

In parallel, analysis of other organs showed that the increased CD45^+^ immune cell infiltration was largely confined to the eWAT, with no statistically significant changes detected in other tissues, although the uterus showed a tendency toward higher CD45^+^ area in HFD-fed animals (Supplementary Fig. S1D). Consistent with these organ-specific effects, we did not detect measurable effects of HFD on systemic immune cell composition in the blood (Fig. 1E; Supplementary Fig. S1E) nor levels of circulating inflammatory markers (Fig. 1F). These data indicate that HFD-induced obesity in this model drives tissue remodeling and immune infiltration specifically in eWAT, with no detectable inflammatory changes in other tissues examined and no systemic cytokine elevation.

Thus, we generated a large-scale cohort for assessing interaction between HFD-induced obesity and *Trp53*^*R270H/+*^ induced cancer. These data indicate that HFD exposure successfully triggered a chronic obesogenic response in our cohorts and that the *Trp53*^*R270H/+*^ mutation does not affect the physiologic response to HFD, which is evident in the eWAT and circulating plasma lipid profile, thereby validating the metabolic challenge for subsequent tumor analysis.

### HFD does not alter tumor-free survival in *Trp53*^*R270H/+*^ mice

**Figure 2.**
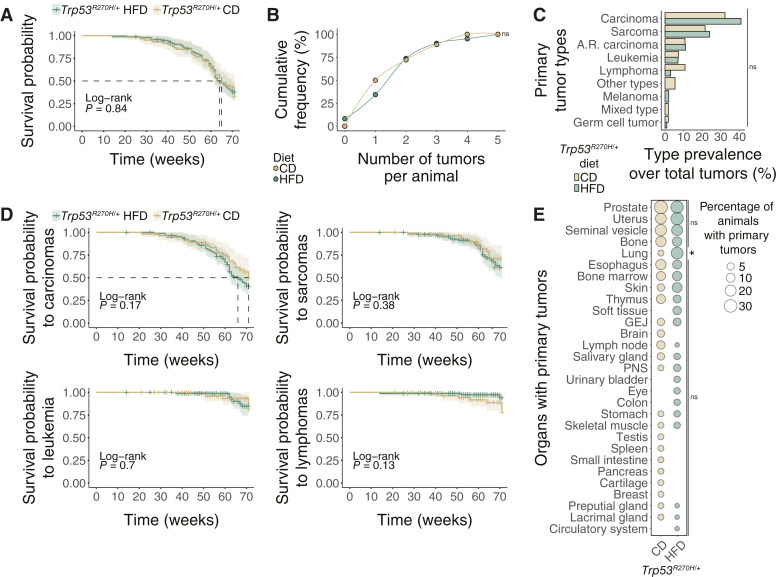
HFD does not alter survival, burden, or overall spectrum in *Trp53*^*R270H/+*^ mice. **A,** Kaplan–Meier survival probability for *Trp53*^*R270H/+*^ animals under CD (beige) or HFD (green) diet. No significant differences were observed with a log-rank test, *P* = 0.84. *N* = 155 animals (60 CD vs. 95 HFD, pooled female and male data). **B,** Cumulative distribution of tumor burden (number of tumors per animal) in *Trp53*^*R270H/+*^ animals fed with CD or HFD. No significant differences (ns) were observed according to an asymptotic two-sided, two-sample Kolmogorov–Smirnov test (D = 0.020408, *P* value = 1). *N* = 97 animals (61 HFD vs. 36 CD, pooled female and male data). **C,** Prevalence of each tumor type over the total number of tumors in CD or HFD-fed *Trp53*^*R270H/+*^ animals. *N* = 168 tumors (108 tumors in HFD- vs. 60 in CD-fed animals, pooled female and male data). No significant differences (ns) were detected using Poisson regression with a log offset for time until euthanasia. A.R., age-related. **D,** Kaplan–Meier survival probability for *Trp53*^*R270H/+*^ animals under CD (beige) or HFD (green) diet for the different tumor types, as specified in the *y*-axis. No significant differences were observed with a log-rank test: *P* = 0.17 (carcinomas), 0.38 (sarcomas), 0.7 (leukemia), and 0.13 (lymphomas). *N* = 155 animals (60 CD vs. 95 HFD, pooled female and male data). **E,** Distribution of primary tumor sites in *Trp53*^*R270H/+*^ mice fed CD or HFD. Bubble size indicates the percentage of animals with tumors in each organ. Poisson regression with a log offset for time until euthanasia. To control the false-positive rate, we considered a tissue significant only if its 95% confidence interval (CI) for the odds ratio lay entirely above 1.1 or entirely below 0.9. For lungs, *P* = 0.0357328, estimate = 2.1736567, and 95% CI, 0.1449047–4.2024087. ns, nonsignificant. *N* = 92 animals (21 females and 15 males under CD and 33 females and 23 males under HFD). GEJ, gastroesophageal junction; PNS, peripheral nervous system. *, *P* < 0.05.

We next measured the effect of HFD feeding on tumor onset in *Trp53*^*R270H/+*^ mice. Despite ∼1 year of obesogenic diet and associated obesity, we found no difference in survival probability between the HFD-fed and control CD-fed *Trp53*^*R270H/+*^ animals. Both groups showed nearly identical median survival times of 64 to 65 weeks (Fig. 2A). No evidence of confounding heterogeneity effects (e.g., altered shape of the Kaplan–Meier curve) was observed.

The absence of dietary effect was consistent across both sexes (Supplementary Fig. S2A–S2F) and reproducible in both independent cohorts that were run approximately 2 years apart (Supplementary Fig. S2B). The only exception was a nonsignificant trend in carcinoma-specific survival in females (*P* = 0.063; Supplementary Fig. S2F), an observation that may warrant follow-up in a study specifically powered for this comparison. The first (discovery) cohort was run and analyzed before the COVID-19 pandemic, while the second (validation) cohort afterward. Each cohort included at least 14 animals per genotype, sex, and diet. The animals from the two cohorts were also derived from distinct sets of breeding pairs (arguing against unexpected parental or litter-specific anomalies) and received distinct manufacturing batches of diets. Together, these findings demonstrate a robust and reproducible lack of impact of chronic HFD-induced obesity on overall survival in *Trp53*^*R270H/+*^ mice. Thus, overall survival of *Trp53*^*R270H/+*^ is refractory to HFD-induced obesity.

### HFD shows minimal impact on tumor burden, prevalence, and spectrum in *Trp53*^*R270H/+*^ mice

True to the model, most *Trp53*^*R270H/+*^ animals exhibited high overall tumor burden with multiple primary tumors per animal. However, HFD-fed animals showed no difference in tumor burden compared with animals in a standard control diet (Fig. 2B). This observation held for independent analyses of female and male animals (Supplementary Fig. S2C) and across discovery and validation cohorts (Supplementary Fig. S2D).

LFS and the *Trp53*^*R270H/+*^ model are characterized by heightened cancer incidence across a range of tumor types and a wide range of targeted tissues. Where sufficiently powered, we also tested for HFD-induced changes in tumor type. Consistent with literature (83), the majority of tumors were carcinomas and sarcomas, followed by leukemias and lymphomas (Fig. 2C). No evidence of altered tumor spectrum was found comparing HFD and CD *Trp53*^*R270H/+*^ animals. Consistent with this, JSD analysis confirmed a low divergence in cancer-type prevalence between diet groups (JSD = 0.06), indicating substantial overall similarity in tumor spectrum (Fig. 2C). Again, these observations were true for both female and male comparisons (Supplementary Fig. S2E). Consistent with this finding, we also did not observe any significant survival differences when stratifying animals by major tumor types, including carcinomas, sarcomas, or lymphoid malignancies (Fig. 2D; Supplementary Fig. S2F).

### HFD shows selective impact on the development of lung tumors in *Trp53*^*R270H/+*^ mice

Primary sites for tumor emergence in *Trp53*^*R270H/+*^ animals included the reproductive system (prostate, uterus, and seminal vesicles), bone and bone marrow, lungs, esophagus, skin, thymus, and soft tissues. Comparisons across tissue of origin showed a modest divergence in tumor site distribution between HFD- and CD-fed animals (JSD = 0.17), indicating some heterogeneity at the anatomic level. The only site reaching statistical significance was the lung, where HFD feeding induced a measurable increase in tumor incidence in *Trp53*^*R270H/+*^ animals (Poisson regression *P* = 0.0357328, SGPV = 0; Fig. 2E). This effect was evident when tumor prevalence was assessed between females and males, with animals of both sexes displaying a marked increase in lung bronchoalveolar carcinoma (BAC) under HFD-feeding conditions (Supplementary Fig. S3A and S3B). Lung tumors were absent in male *Trp53*^*R270H/+*^ animals fed a control diet but constituted more than 10% of the tumor spectrum of HFD-fed mice (Supplementary Fig. S3B).

Molecular analysis of lung tumors revealed HFD-specific differences in the *Trp53* locus and expressed protein. First, we found lower tumor-to-normal p53 immunofluorescence ratio in lungs from HFD-fed animals compared with controls. Crucially, we did not observe these diet-specific differences in p53 protein levels in any other tumor-affected tissues. This was true even in tissues like the uterus, where there was a substantial heterogeneity in p53 protein expression within the tumors of both HFD- and CD-fed animals (Supplementary Fig. S3C). Importantly, the p53 antibody we used to detect total p53 protein does not distinguish between WT and mutant forms. Second, ddPCR analysis showed a trend toward an increased mutant-to-WT ratio for the *Trp53* alleles in lung tumor relative to contralateral normal tissue from HFD-fed animals, suggesting a potential loss-of-heterozygosity (LOH) event at this locus under HFD conditions (Supplementary Fig. S3D).

No significant differences in tumor classification were observed across groups when comparing the distribution of benign versus malignant tumors by diet and sex (Supplementary Fig. S3E).

These results strongly suggest that the HFD-induced changes in the *Trp53* allelic and protein profiles are specific to the lung environment. The lung CD tumor sample represents a single available case, reflecting the limited number of animals and tissue availability for this tumor type under these conditions; nonetheless, the observed trend is consistent with the overall pattern of reduced p53 protein levels in HFD-fed animals.

Representative H&E images for all observed tumor types are included in Supplementary Fig. S4A and S4β. All reported tumor sites represent histologically confirmed primary sites of origin, excluding disseminated or metastatic lesions. Representative examples of metastases, consistent with expected patterns for their respective primary tumors, are presented in Supplementary Fig. S5A–S5s. A systematic evaluation of metastasis was beyond the scope of this study.

In summary, *Trp53*^*R270H/+*^-dependent cancers are largely refractory to the effects of HFD-induced obesity, with the lung representing a selective exception where both incidence and *Trp53* molecular features exhibit diet sensitivity.

## Discussion

Overall, our data provide an example in which, under the conditions tested, obesity and HFD-induced metabolic changes have limited impact on tumorigenesis and cancer outcomes. Our results suggest that chronic HFD-induced obesity does not alter total tumor burden in *Trp53*^*R270H/+*^ mice. *Trp53*^*R270H/+*^-dependent cancers are largely refractory to the effects of HFD-induced obesity, with the lung representing a selective exception in which both incidence and *Trp53* molecular features exhibit sensitivity to the diet. The similarity in tumor onset and severity in *Trp53*^*R270H/+*^ mice regardless of diet, as well as the lung-specific effects, are thus complementary observations. Together, the data illustrate the tissue-specific nature of HFD–obesity interactions in this model.

Despite extensive evidence linking obesity to heightened cancer risk through mechanisms such as chronic inflammation, insulin resistance, and altered hormonal signaling (69, 103), our experiments indicate that HFD-induced obesity does not significantly alter or worsen tumorigenesis in the *Trp53*^*R270H/+*^ mouse model, with the one exception being the increased development of lung BACs. One possible explanation for this lack of impact on tumor onset and burden is that the effects of obesity-associated inflammation (especially sterile inflammation) might depend on additional physiologic stressors (e.g., oxidative stress, tissue hypoxia, or subclinical infections) that were absent in our controlled conditions (104). Supporting this hypothesis, certain dietary fats have been shown to induce complement activation and intestinal tumorigenesis independent of obesity, emphasizing dietary composition and inflammation rather than obesity itself as the critical factor (105). These observations align with our previous findings, in which *Trim28*^*+/D9*^ heavy mice exhibited increased adiposity without accompanying inflammatory responses or heightened cancer incidence, suggesting that metabolic health rather than adiposity *per se* may determine cancer outcomes (72, 97). Moreover, the genetic context of the *Trp53*^*R270H/+*^ mutation itself may alter the tissue microenvironment, potentially buffering inflammatory disruptions typically driven by obesity. Multiple studies identify WT p53 as a key regulator of adipose tissue inflammation, immune cell infiltration, and adipokine signaling, influencing adipocyte–macrophage cross-talk, insulin sensitivity, and sterile inflammation (106–109). Indeed, adipose tissue–specific deletion of *Trp53* (*Trp53*^*fl/fl*^; *Fabp4-Cre*) has been shown to normalize insulin glucose tolerance and cytokine misexpression in obesity-prone agouti mice (Ay) as well as correlate with decreased senescence-like features (110).

Another factor that may affect our results is the contribution from WT p53. WT p53 has been shown to regulate energy metabolism, glycolysis, oxidative phosphorylation, and fatty acid oxidation, which could counterbalance the adverse metabolic and inflammatory profiles associated with high-fat feeding (98, 111, 112). Consistent with these observations and in line with related metabolic changes reported in LFS (113), partial inhibition of fatty acid oxidation has previously delayed tumor onset in *Trp53*^*R270H/+*^ mice (114–116). Collectively, these findings suggest that the metabolic and immune landscape induced by the *Trp53*^*R270H*^ mutation could counteract or buffer the inflammatory and metabolic disruptions typically associated with chronic obesity. In support of this interpretation, our data show that although HFD triggered strong metabolic remodeling and localized immune infiltration restricted to eWAT, these changes did not translate into systemic immune activation or enhanced tumorigenesis. This observation contrasts with the systemic inflammation typically observed in metabolically unhealthy obesity in humans and is consistent with the well-documented tendency of C57BL/6-based mouse models to exhibit localized rather than systemic inflammatory responses to HFD.

Importantly, although our data clearly show a lack of effect of HFD and HFD-induced obesity on mutant p53-dependent tumorigenesis, they do not rule out all potential associations between obesities and R270H-driven cancer. Our limited understanding of obesity-cancer associations is exacerbated by a lack of knowledge about the heterogeneity of human obesity itself. Our work (71–73, 97) and that of others (117–119) have highlighted that obesity is an umbrella term for what are a collection of disorders. Heterogeneous clinical presentations include metabolically “healthy” and “unhealthy” obesities, types A and B obesities (73), and a complex intersection of clinically relevant heterogeneity in body size, shape, and composition (120–122). Although our work highlights a negative interaction between HFD-induced obesity and *Trp53*^*R270H/+*^ cancers, this does not mean that other obesity “endotypes” (i.e., etiologically distinct obesity subtypes) will not exhibit a strong interaction. Testing causal links between multiple obesity subtypes and their respective dietary associations, across myriad cancer types, comprises a state-of-the-art challenge for the field (3).

Although we observed no significant survival differences between HFD-fed and control-fed *Trp53*^*R270H/+*^ mice (Fig. 2A), it remains possible that differences in tumor growth kinetics or aggressiveness exist but were not detectable due to the endpoint-based nature of our experimental design. It remains possible that effects of HFD-induced obesity on tumorigenesis or survival could emerge in a subset of animals with extended aging beyond this timeframe. Moreover, our assessment relied primarily on endpoint analysis and histologic evaluation rather than continuous, longitudinal monitoring of tumor growth dynamics.

An additional limitation relates to differences in dietary composition beyond macronutrient content. The control chow used in this study contains soy and other phytoestrogen-rich ingredients, whereas the purified HFD is largely devoid of phytoestrogens and antioxidants. Dietary phytoestrogens have been shown to exert tissue-specific pro- or anti-tumorigenic effects in mouse models, and thus we cannot fully exclude the contribution of these dietary compounds to tumor growth (123–130). However, we note that tissues with the best-documented phytoestrogen sensitivity in mouse models (prostate, colon, uterus, and bladder) show no differential tumor incidence between CD and HFD animals in our cohort. In addition, the growth of tumors with no established estrogen sensitivity, such as sarcomas, leukemias, and lymphomas, were similar *Trp53*^*R270H/+*^ mice regardless of diet. The absence of any diet-dependent difference across this full spectrum of estrogen-sensitive and -insensitive tumor types argues against phytoestrogen contributing to tumor growth in our model.

Although *Trp53*^*R270H/+*^ mice recapitulate many clinical features of human LFS, important differences in cancer spectrum exist. Most notably, these mice exhibit a significantly lower frequency of breast cancer compared with human patients with LFS, who commonly present breast malignancies at an early age (83, 131, 132). This discrepancy likely arises from differences in species-specific mammary gland biology, hormonal regulation, and genetic background interactions, factors previously reported to profoundly influence tumor susceptibility in p53-mutant models (83, 133–135). Thus, caution is necessary when extrapolating findings from mouse models directly to human clinical scenarios, particularly regarding tumor types with known species-specific variation. Our study also documented representative examples of metastatic lesions across several tumor types, each displaying patterns consistent with their expected primary origins. However, a systematic assessment of metastatic potential was beyond the scope of this study. Comprehensive evaluation of metastasis typically requires advanced imaging and multilevel histopathologic sampling to resolve local versus distant spread, which are not compatible with the large cohort size and experimental design used in this study. It is also important to emphasize that survival data from murine cancer models differ substantially from human cancer survival, primarily due to the absence of therapeutic interventions. Unlike human patients who routinely receive treatment upon diagnosis, mice in these experimental cohorts were untreated, limiting the translational relevance of survival outcomes. Thus, interpretations of our survival results should remain cautious and primarily focused on intrinsic tumor progression rather than clinical prognosis. Collectively, our study captures the overall landscape of primary tumorigenesis in the context of HFD feeding, whereas the impact of HFD-driven obesity on metastasis and therapeutic efficacy remains an avenue for future investigation.

Our molecular analyses of lung tumors suggest that, whereas most tissues remain refractory to HFD, the lung represents a unique site where HFD feeding alters the allelic balance or expression of mutant p53. An important technical consideration is that the p53 antibody we used in this study cannot differentiate between WT and mutant forms of p53. This has direct implications for interpreting our immunofluorescence data. Under CD conditions, the elevated tumor-to-normal p53 signal is consistent with accumulation of total p53, which may reflect canonical stabilization of the missense mutant protein, WT p53 accumulation in response to cellular stress, or a combination of both. Under HFD conditions, the trend toward an increased mutant-to-WT allelic ratio suggests progressive loss of the WT allele (84). In this context, the observed reduction in total p53 protein signal may not reflect destabilization of the mutant protein per se but rather the loss of the WT protein contribution to the total detectable signal, an interpretation consistent with the LOH trend and the heterozygous nature of the model (102, 115–120). Taken together, these data suggest that HFD-induced metabolic alterations in the lung microenvironment selectively favor loss of the WT allele (111, 112), contributing to a state of functional p53 loss that may underlie the observed diet-dependent lung tumor phenotypes. Whether the mutant protein itself is stabilized or destabilized under these conditions could not be resolved in the current study and would require allele-specific protein quantification. These context-dependent molecular dynamics, specifically in the lung environment, warrant further investigation.

Given the obesity epidemic in Western societies, the impact of diet on cancer development and progression is highly relevant to human health. Recent literature contains select reports that question the causal association between BMI and reduced cancer survival (8, 17–26). Our study adds to this growing body of evidence, supporting epidemiologic observations that obesity does not uniformly accelerate tumorigenesis and may have neutral or even context-dependent effects, particularly in *p53*-mutant settings (8, 28, 136–138). Beyond p53, this study identifies a new model for examining long-term tumor and host dynamics (genetic, transcriptional, immune, epigenetic, and metabolic) in contexts of combined cancer susceptibility and metabolic disease evolution. The uncoupling of dietary effects from an altered cancer trajectory affords unique interdisciplinary opportunities for understanding chronic cancer and metabolic disease coevolution.

## Supplementary Material

Supplementary Figure 1 Longitudinal body and fat mass measurements, adipose and tissue immune staining, lymphocyte gating strategy, and genotype/diet comparisons across female and male mice under chow or high-fat diet conditions.

Supplementary Figure 2 Survival analyses, tumor burden distributions, tumor-type prevalence, and tumor-specific survival comparisons in female and male Trp53R270H/+ mice under chow or high-fat diet conditions, including pre- and post-COVID cohorts.

Supplementary Figure 3 Organ and tumor-type distributions, tumor malignancy frequencies, TRP53 staining analyses, and mutant-to-wild-type Trp53 allele ratios in tumors and matched normal tissues from female and male mice under chow or high-fat diet conditions.

Supplementary Figure 4 Representative histological images of diverse tumor types identified in the study, including various carcinomas, sarcomas, and lymphomas, with specific examples of metastatic lesions in the lungs and liver across different genotype and diet cohorts.

Supplementary Figure 5 Comparison of tumor-free survival and overall survival curves between Trp53(R270H/+) and wild-type mice, stratified by sex and diet, along with quantitative analysis of tumor multiplicity and the distribution of primary versus metastatic lesions.

Supplementary Table 1 Detailed comparison of energy distribution, macronutrient mass composition, and micronutrient profiles between the standard Purina 5021 chow and the D12492 high-fat purified diet used in the study.

Supplementary Table 2 Comprehensive individual mouse records including genotype, sex, and diet assignments, longitudinal body weight and composition measurements, age at sacrifice, and detailed pathological assessments of tumor incidence and metastatic status.

Supplementary Table 3 Nucleotide sequences for the forward and reverse primers used for the PCR-based genotyping of the Trp53 alleles in the experimental mouse cohort.

Supplementary Table 4 Summary of the thermal cycling parameters, including temperatures and durations for denaturation, annealing, and extension phases, used for the PCR amplification of the Trp53 gene.

Supplementary Table 5 Details of the restriction enzyme digestion protocol, including specific incubation temperatures and times for the MslI enzyme and subsequent heat inactivation, used for Trp53 allele discrimination.

Supplementary Table 6 Sequences for the forward and reverse primers and allele-specific probes used for the quantitative PCR-based discrimination between wild-type and mutant Trp53 alleles.

## Data Availability

The MS lipidomics data have been deposited to the MassIVE repository (ucsd.edu) with the dataset identifier MSV000101772. Other data generated in this study are provided in the supplementary files or are available upon request to the corresponding author.

## References

[1] Ward ZJ , Bleich SN, Long MW, Gortmaker SL. Association of body mass index with health care expenditures in the United States by age and sex. PLoS One 2021;16:e0247307. 33760880 10.1371/journal.pone.0247307 PMC7990296

[2] Renehan AG , Tyson M, Egger M, Heller RF, Zwahlen M. Body-mass index and incidence of cancer: a systematic review and meta-analysis of prospective observational studies. Lancet 2008;371:569–78. 18280327 10.1016/S0140-6736(08)60269-X

[3] Bhaskaran K , Douglas I, Forbes H, dos-Santos-Silva I, Leon DA, Smeeth L. Body-mass index and risk of 22 specific cancers: a population-based cohort study of 5 to 24 million UK adults. Lancet 2014;384:755–65. 25129328 10.1016/S0140-6736(14)60892-8 PMC4151483

[4] Moore LL , Chadid S, Singer MR, Kreger BE, Denis GV. Metabolic health reduces risk of obesity-related cancer in Framingham study adults. Cancer Epidemiol Biomarkers Prev 2014;23:2057–65. 25012997 10.1158/1055-9965.EPI-14-0240 PMC4184957

[5] Mahamat-saleh Y , Aune D, Freisling H, Hardikar S, Jaafar R, Rinaldi S, . Association of metabolic obesity phenotypes with risk of overall and site-specific cancers: a systematic review and meta-analysis of cohort studies. Br J Cancer 2024;131:1480–95. 39317703 10.1038/s41416-024-02857-7 PMC11519895

[6] Lauby-Secretan B , Scoccianti C, Loomis D, Grosse Y, Bianchini F, Straif K. Body fatness and cancer–viewpoint of the IARC Working Group. N Engl J Med 2016;375:794–8. 27557308 10.1056/NEJMsr1606602 PMC6754861

[7] Marinac CR , Birmann BM, Lee IM, Rosner BA, Townsend MK, Giovannucci E, . Body mass index throughout adulthood, physical activity, and risk of multiple myeloma: a prospective analysis in three large cohorts. Br J Cancer 2018;118:1013–9. 29527008 10.1038/s41416-018-0010-4 PMC5931105

[8] Petrelli F , Cortellini A, Indini A, Tomasello G, Ghidini M, Nigro O, . Association of obesity with survival outcomes in patients with cancer: a systematic review and meta-analysis. JAMA Netw Open 2021;4:e213520. 33779745 10.1001/jamanetworkopen.2021.3520 PMC8008284

[9] Ligibel JA , Alfano CM, Courneya KS, Demark-Wahnefried W, Burger RA, Chlebowski RT, . American Society of Clinical Oncology position statement on obesity and cancer. J Clin Oncol 2014;32:3568–74. 25273035 10.1200/JCO.2014.58.4680 PMC4979237

[10] Rock CL , Doyle C, Demark-Wahnefried W, Meyerhardt J, Courneya KS, Schwartz AL, . Nutrition and physical activity guidelines for cancer survivors. CA Cancer J Clin 2012;62:242–74. 10.3322/caac.21142 22539238

[11] Senkus E , Kyriakides S, Penault-Llorca F, Poortmans P, Thompson A, Zackrisson S, . Primary breast cancer: ESMO Clinical Practice Guidelines for diagnosis, treatment and follow-up. Ann Oncol 2013;24:vi7–23. 23970019 10.1093/annonc/mdt284

[12] Font-Burgada J , Sun B, Karin M. Obesity and cancer: the oil that feeds the flame. Cell Metab 2016;23:48–62. 26771116 10.1016/j.cmet.2015.12.015

[13] Perry RJ , Shulman GI. Mechanistic links between obesity, insulin, and cancer. Trends Cancer 2020;6:75–8. 32061306 10.1016/j.trecan.2019.12.003 PMC7214048

[14] Li R , Grimm SA, Mav D, Gu H, Djukovic D, Shah R, . Transcriptome and DNA methylome analysis in a mouse model of diet-induced obesity predicts increased risk of colorectal cancer. Cell Rep 2018;22:624–37. 29346762 10.1016/j.celrep.2017.12.071 PMC5793878

[15] Calabrese C , Miserocchi G, De Vita A, Spadazzi C, Cocchi C, Vanni S, . Lipids and adipocytes involvement in tumor progression with a focus on obesity and diet. Obes Rev 2024;25:e13833. 39289899 10.1111/obr.13833

[16] Engin AB , Engin A. Next-cell hypothesis: mechanism of obesity-associated carcinogenesis. Adv Exp Med Biol 2024;1460:727–66. 39287871 10.1007/978-3-031-63657-8_25

[17] Amptoulach S , Gross G, Kalaitzakis E. Differential impact of obesity and diabetes mellitus on survival after liver resection for colorectal cancer metastases. J Surg Res 2015;199:378–85. 26115811 10.1016/j.jss.2015.05.059

[18] Banack HR , Kaufman JS. The obesity paradox: understanding the effect of obesity on mortality among individuals with cardiovascular disease. Prev Med 2014;62:96–102. 24525165 10.1016/j.ypmed.2014.02.003

[19] Brunner AM , Sadrzadeh H, Feng Y, Drapkin BJ, Ballen KK, Attar EC, . Association between baseline body mass index and overall survival among patients over age 60 with acute myeloid leukemia. Am J Hematol 2013;88:642–6. 23619915 10.1002/ajh.23462 PMC4214755

[20] Hakimi AA , Furberg H, Zabor EC, Jacobsen A, Schultz N, Ciriello G, . An epidemiologic and genomic investigation into the obesity paradox in renal cell carcinoma. J Natl Cancer Inst 2013;105:1862–70. 24285872 10.1093/jnci/djt310 PMC3866155

[21] Hines RB , Shanmugam C, Waterbor JW, McGwin G Jr, Funkhouser E, Coffey CS, . Effect of comorbidity and body mass index on the survival of African-American and Caucasian patients with colon cancer. Cancer 2009;115:5798–806. 19937953 10.1002/cncr.24598 PMC2795032

[22] Mayeda ER , Glymour MM. The obesity paradox in survival after cancer diagnosis: tools for evaluation of potential bias. Cancer Epidemiol Biomarkers Prev 2017;26:17–20. 28069728 10.1158/1055-9965.EPI-16-0559 PMC5858690

[23] Navarro WH , Loberiza FR Jr, Bajorunaite R, van Besien K, Vose JM, Lazarus HM, . Effect of body mass index on mortality of patients with lymphoma undergoing autologous hematopoietic cell transplantation. Biol Blood Marrow Transplant 2006;12:541–51. 16635789 10.1016/j.bbmt.2005.12.033

[24] Park Y , Peterson LL, Colditz GA. The plausibility of obesity paradox in cancer—point. Cancer Res 2018;78:1898–903. 29654151 10.1158/0008-5472.CAN-17-3043 PMC5903573

[25] Parker AS , Lohse CM, Cheville JC, Thiel DD, Leibovich BC, Blute ML. Greater body mass index is associated with better pathologic features and improved outcome among patients treated surgically for clear cell renal cell carcinoma. Urology 2006;68:741–6. 17070345 10.1016/j.urology.2006.05.024

[26] Schlesinger S , Siegert S, Koch M, Walter J, Heits N, Hinz S, . Postdiagnosis body mass index and risk of mortality in colorectal cancer survivors: a prospective study and meta-analysis. Cancer Causes Control 2014;25:1407–18. 25037235 10.1007/s10552-014-0435-x

[27] Lysaght J , Conroy MJ. The multifactorial effect of obesity on the effectiveness and outcomes of cancer therapies. Nat Rev Endocrinol 2024;20:701–14. 39313571 10.1038/s41574-024-01032-5

[28] Lennon H , Sperrin M, Badrick E, Renehan AG. The obesity paradox in cancer: a review. Curr Oncol Rep 2016;18:56. 27475805 10.1007/s11912-016-0539-4 PMC4967417

[29] Tuck CJ , De Palma G, Takami K, Brant B, Caminero A, Reed DE, . Nutritional profile of rodent diets impacts experimental reproducibility in microbiome preclinical research. Sci Rep 2020;10:17784. 33082369 10.1038/s41598-020-74460-8 PMC7575541

[30] Speakman JR . Use of high-fat diets to study rodent obesity as a model of human obesity. Int J Obes 2019;43:1491–2. 10.1038/s41366-019-0363-7 30967607

[31] Lien EC , Vander Heiden MG. A framework for examining how diet impacts tumour metabolism. Nat Rev Cancer 2019;19:651–61. 31530936 10.1038/s41568-019-0198-5

[32] Hariri N , Thibault L. High-fat diet-induced obesity in animal models. Nutr Res Rev 2010;23:270–99. 20977819 10.1017/S0954422410000168

[33] Kunkemoeller B , Prendeville H, McIntyre C, Temesgen A, Loftus RM, Yao C, . The source of dietary fat influences anti-tumour immunity in obese mice. Nat Metab 2025;7:1630–45. 40715760 10.1038/s42255-025-01330-w PMC12373505

[34] Showalter MR , Nonnecke EB, Linderholm AL, Cajka T, Sa MR, Lönnerdal B, . Obesogenic diets alter metabolism in mice. PLoS One 2018;13:e0190632. 29324762 10.1371/journal.pone.0190632 PMC5764261

[35] Lang P , Hasselwander S, Li H, Xia N. Effects of different diets used in diet-induced obesity models on insulin resistance and vascular dysfunction in C57BL/6 mice. Sci Rep 2019;9:19556. 31862918 10.1038/s41598-019-55987-x PMC6925252

[36] Jeong Y-J , Rogers TJ, Anderson CE, Lien EC. Tumor lipid metabolism: a mechanistic link between diet and cancer progression. Curr Opin Biotechnol 2023;84:102993. 37716318 10.1016/j.copbio.2023.102993 PMC10872979

[37] de Moura e Dias M , dos Reis SA, da Conceição LL, de Oliveira Sediyama CMN, Pereira SS, de Oliveira LL, . Diet-induced obesity in animal models: points to consider and influence on metabolic markers. Diabetol Metab Syndr 2021;13:32. 33736684 10.1186/s13098-021-00647-2 PMC7976703

[38] Lee Y-Y , Tang TK, Phuah ET, Karim NAA, Alitheen NBM, Tan CP, . Structural difference of palm based Medium- and Long-Chain Triacylglycerol (MLCT) further reduces body fat accumulation in DIO C57BL/6J mice when consumed in low fat diet for a mid-term period. Food Res Int 2018;103:200–7. 29389606 10.1016/j.foodres.2017.10.022

[39] Galbo T , Perry RJ, Jurczak MJ, Camporez JPG, Alves TC, Kahn M, . Saturated and unsaturated fat induce hepatic insulin resistance independently of TLR-4 signaling and ceramide synthesis in vivo. Proc Natl Acad Sci U S A 2013;110:12780–5. 23840067 10.1073/pnas.1311176110 PMC3732992

[40] Kennedy A , Martinez K, Chuang C-C, LaPoint K, McIntosh M. Saturated fatty acid-mediated inflammation and insulin resistance in adipose tissue: mechanisms of action and implications. J Nutr 2009;139:1–4. 19056664 10.3945/jn.108.098269

[41] Sears B , Perry M. The role of fatty acids in insulin resistance. Lipids Health Dis 2015;14:121. 26415887 10.1186/s12944-015-0123-1 PMC4587882

[42] Griel AE , Ruder EH, Kris-Etherton PM. The changing roles of dietary carbohydrates. Arterioscler Thromb Vasc Biol 2006;26:1958–65. 16794219 10.1161/01.ATV.0000233384.97125.bd

[43] Takeuchi T , Kubota T, Nakanishi Y, Tsugawa H, Suda W, Kwon ATJ, . Gut microbial carbohydrate metabolism contributes to insulin resistance. Nature 2023;621:389–95. 37648852 10.1038/s41586-023-06466-x PMC10499599

[44] Zhang S , Xiao X, Yi Y, Wang X, Zhu L, Shen Y, . Tumor initiation and early tumorigenesis: molecular mechanisms and interventional targets. Signal Transduct Target Ther 2024;9:149. 38890350 10.1038/s41392-024-01848-7 PMC11189549

[45] Hasan N , Yazdanpanah O, Khaleghi B, Benjamin DJ, Kalebasty AR. The role of dietary sugars in cancer risk: a comprehensive review of current evidence. Cancer Treat Res Commun 2025;43:100876. 39922003 10.1016/j.ctarc.2025.100876

[46] Olmedo M , Santiago S, Romanos-Nanclares A, Aramendia-Beitia JM, Sanchez-Bayona R, Bes-Rastrollo M, . Dietary carbohydrate quality index and incidence of obesity-related cancers in the “Seguimiento Universidad De Navarra” (SUN) prospective cohort. Eur J Nutr 2024;63:2449–58. 38814364 10.1007/s00394-024-03438-3 PMC11490434

[47] Charles-Messance H , Mitchelson KAJ, De Marco Castro E, Sheedy FJ, Roche HM. Regulating metabolic inflammation by nutritional modulation. J Allergy Clin Immunol 2020;146:706–20. 32841652 10.1016/j.jaci.2020.08.013

[48] Armet AM , Deehan EC, Thöne JV, Hewko SJ, Walter J. The effect of isolated and synthetic dietary fibers on markers of metabolic diseases in human intervention studies: a systematic review. Adv Nutr 2020;11:420–38. 31342059 10.1093/advances/nmz074 PMC7442353

[49] Kim K , Chang Y. Association of dietary fiber intake with metabolic syndrome among adult cancer survivors: a population-based cross-sectional study. Sci Rep 2021;11:11794. 34083714 10.1038/s41598-021-91312-1 PMC8175362

[50] Maguolo A , Gabbianelli R, Maffeis C. Micronutrients in early life and offspring metabolic health programming: a promising target for preventing non-communicable diseases. Eur J Clin Nutr 2023;77:1105–12. 37604969 10.1038/s41430-023-01333-4

[51] Li J , Wu H, Liu Y, Yang L. High fat diet induced obesity model using four strainsof mice: Kunming, C57BL/6, BALB/c and ICR. Exp Anim 2020;69:326–35. 32188837 10.1538/expanim.19-0148 PMC7445062

[52] Griffin LE , Essenmacher L, Racine KC, Iglesias-Carres L, Tessem JS, Smith SM, . Diet-induced obesity in genetically diverse collaborative cross mouse founder strains reveals diverse phenotype response and amelioration by quercetin treatment in 129S1/SvImJ, PWK/EiJ, CAST/PhJ, and WSB/EiJ mice. J Nutr Biochem 2021;87:108521. 33039581 10.1016/j.jnutbio.2020.108521

[53] Bachmann AM , Morel JD, El Alam G, Rodríguez-López S, Imamura de lima T, Goeminne LJ, . Genetic background and sex control the outcome of high-fat diet feeding in mice. iScience 2022;25:104468. 35677645 10.1016/j.isci.2022.104468 PMC9167980

[54] Montgomery MK , Hallahan NL, Brown SH, Liu M, Mitchell TW, Cooney GJ, . Mouse strain-dependent variation in obesity and glucose homeostasis in response to high-fat feeding. Diabetologia 2013;56:1129–39. 23423668 10.1007/s00125-013-2846-8

[55] Smoczek M , Vital M, Wedekind D, Basic M, Zschemisch NH, Pieper DH, . A combination of genetics and microbiota influences the severity of the obesity phenotype in diet-induced obesity. Sci Rep 2020;10:6118. 32273571 10.1038/s41598-020-63340-w PMC7145845

[56] Marei WFA , Smits A, Mohey-Elsaeed O, Pintelon I, Ginneberge D, Bols PEJ, . Differential effects of high fat diet-induced obesity on oocyte mitochondrial functions in inbred and outbred mice. Sci Rep 2020;10:9806. 32555236 10.1038/s41598-020-66702-6 PMC7299992

[57] Dreux V , Lefebvre C, Breemeersch CE, Salaün C, Bôle-Feysot C, Guérin C, . Sex-dependent effects of a high-fat diet on the hypothalamic response in mice. Biol Sex Differ 2025;16:17. 40001261 10.1186/s13293-025-00699-3 PMC11854408

[58] Parks BW , Nam E, Org E, Kostem E, Norheim F, Hui S, . Genetic control of obesity and gut microbiota composition in response to high-fat, high-sucrose diet in mice. Cell Metab 2013;17:141–52. 23312289 10.1016/j.cmet.2012.12.007 PMC3545283

[59] West DB , Waguespack J, McCollister S. Dietary obesity in the mouse: interaction of strain with diet composition. Am J Physiol 1995;268:R658–65. 7900908 10.1152/ajpregu.1995.268.3.R658

[60] Buchner DA , Burrage LC, Hill AE, Yazbek SN, O’Brien WE, Croniger CM, . Resistance to diet-induced obesity in mice with a single substituted chromosome. Physiol Genomics 2008;35:116–22. 18628339 10.1152/physiolgenomics.00033.2008 PMC2536825

[61] Burrage LC , Baskin-Hill AE, Sinasac DS, Singer JB, Croniger CM, Kirby A, . Genetic resistance to diet-induced obesity in chromosome substitution strains of mice. Mamm Genome 2010;21:115–29. 20127486 10.1007/s00335-010-9247-9 PMC3831885

[62] Glavas MM , Lee AY, Miao I, Yang F, Mojibian M, O’Dwyer SM, . Developmental timing of high-fat diet exposure impacts glucose homeostasis in mice in a sex-specific manner. Diabetes 2021;70:2771–84. 34544729 10.2337/db21-0310 PMC8660987

[63] Nascimento-Sales M , Fredo-da-Costa I, Borges Mendes ACB, Melo S, Ravache TT, Gomez TGB, . Is the FVB/N mouse strain truly resistant to diet-induced obesity? Physiol Rep 2017;5:e13271. 28483861 10.14814/phy2.13271 PMC5430125

[64] Wang C-Y , Liao JK. A mouse model of diet-induced obesity and insulin resistance. In: Weichhart T, editor. mTOR: methods and protocols. Totowa (NJ): Humana Press; 2012. p. 421–33. 10.1007/978-1-61779-430-8_27 PMC3807094 22125082

[65] Buettner R , Schölmerich J, Bollheimer LC. High-fat diets: modeling the metabolic disorders of human obesity in rodents. Obesity 2007;15:798–808. 17426312 10.1038/oby.2007.608

[66] George V , Tremblay A, Després JP, Leblanc C, Bouchard C. Effect of dietary fat content on total and regional adiposity in men and women. Int J Obes 1990;14:1085–94. 2086500

[67] Saris WHM , Astrup A, Prentice A, Zunft H, Formiguera X, Verboeket-van de Venne W, . Randomized controlled trial of changes in dietary carbohydrate/fat ratio and simple vs complex carbohydrates on body weight and blood lipids: the CARMEN study. Int J Obes 2000;24:1310–8. 10.1038/sj.ijo.0801451 11093293

[68] Tucker LA , Kano MJ. Dietary fat and body fat: a multivariate study of 205 adult females. Am J Clin Nutr 1992;56:616–22. 1414959 10.1093/ajcn/56.4.616

[69] Rohm TV , Meier DT, Olefsky JM, Donath MY. Inflammation in obesity, diabetes, and related disorders. Immunity 2022;55:31–55. 35021057 10.1016/j.immuni.2021.12.013 PMC8773457

[70] Garaulet M , Madrid JA. Chronobiological aspects of nutrition, metabolic syndrome and obesity. Adv Drug Deliv Rev 2010;62:967–78. 20580916 10.1016/j.addr.2010.05.005

[71] Adams KF , Schatzkin A, Harris TB, Kipnis V, Mouw T, Ballard-Barbash R, . Overweight, obesity, and mortality in a large prospective cohort of persons 50 to 71 years old. N Engl J Med 2006;355:763–78. 16926275 10.1056/NEJMoa055643

[72] Dalgaard K , Landgraf K, Heyne S, Lempradl A, Longinotto J, Gossens K, . Trim28 haploinsufficiency triggers Bi-stable epigenetic obesity. Cell 2016;164:353–64. 26824653 10.1016/j.cell.2015.12.025 PMC4735019

[73] Yang C-H , Fagnocchi L, Apostle S, Wegert V, Casaní-Galdón S, Landgraf K, . Independent phenotypic plasticity axes define distinct obesity sub-types. Nat Metab 2022;4:1150–65. 36097183 10.1038/s42255-022-00629-2 PMC9499872

[74] Beyaz S , Mana MD, Roper J, Kedrin D, Saadatpour A, Hong SJ, . High-fat diet enhances stemness and tumorigenicity of intestinal progenitors. Nature 2016;531:53–8. 26935695 10.1038/nature17173 PMC4846772

[75] Park EJ , Lee JH, Yu GY, He G, Ali SR, Holzer RG, . Dietary and genetic obesity promote liver inflammation and tumorigenesis by enhancing IL-6 and TNF expression. Cell 2010;140:197–208. 20141834 10.1016/j.cell.2009.12.052 PMC2836922

[76] Philip B , Roland CL, Daniluk J, Liu Y, Chatterjee D, Gomez SB, . A high-fat diet activates oncogenic Kras and COX2 to induce development of pancreatic ductal adenocarcinoma in mice. Gastroenterology 2013;145:1449–58. 23958541 10.1053/j.gastro.2013.08.018 PMC3873752

[77] Maguire OA , Ackerman SE, Szwed SK, Maganti AV, Marchildon F, Huang X, . Creatine-mediated crosstalk between adipocytes and cancer cells regulates obesity-driven breast cancer. Cell Metab 2021;33:499–512.e6. 33596409 10.1016/j.cmet.2021.01.018 PMC7954401

[78] Labbé DP , Zadra G, Yang M, Reyes JM, Lin CY, Cacciatore S, . High-fat diet fuels prostate cancer progression by rewiring the metabolome and amplifying the MYC program. Nat Commun 2019;10:4358. 31554818 10.1038/s41467-019-12298-z PMC6761092

[79] Xia S , Lin R, Jin L, Zhao L, Kang HB, Pan Y, . Prevention of dietary-fat-fueled ketogenesis attenuates BRAF V600E tumor growth. Cell Metab 2017;25:358–73. 28089569 10.1016/j.cmet.2016.12.010 PMC5299059

[80] Hollstein M , Sidransky D, Vogelstein B, Harris CC. p53 mutations in human cancers. Science 1991;253:49–53. 1905840 10.1126/science.1905840

[81] Li FP , Fraumeni JF Jr. Soft-tissue sarcomas, breast cancer, and other neoplasms. Ann Intern Med 1969;71:747–52. 5360287 10.7326/0003-4819-71-4-747

[82] Guha T , Malkin D. Inherited TP53 mutations and the Li–Fraumeni syndrome. Cold Spring Harbor Perspect Med 2017;7:a026187. 10.1101/cshperspect.a026187 PMC5378014 28270529

[83] Olive KP , Tuveson DA, Ruhe ZC, Yin B, Willis NA, Bronson RT, . Mutant p53 gain of function in two mouse models of Li-Fraumeni syndrome. Cell 2004;119:847–60. 15607980 10.1016/j.cell.2004.11.004

[84] Wood SN . Fast stable restricted maximum likelihood and marginal likelihood estimation of semiparametric generalized linear models. J R Stat Soc Ser B Stat Methodol 2010;73:3–36.

[85] Zeileis A , Hothorn T. Diagnostic checking in regression relationships. R News 2001;2:7–10.

[86] Blume JD , D’Agostino McGowan L, Dupont WD, Greevy RA Jr. Second-generation p-values: improved rigor, reproducibility, & transparency in statistical analyses. PLoS One 2018;13:e0188299. 29565985 10.1371/journal.pone.0188299 PMC5863943

[87] Blume JD , Greevy RA, Welty VF, Smith JR, Dupont WD. An introduction to second-generation p-values. Am Stat 2019;73:157–67.

[88] Drost H-G . Philentropy: information theory and distance quantification with R. J Open Source Softw 2018;3:765.

[89] Bankhead P , Loughrey MB, Fernández JA, Dombrowski Y, McArt DG, Dunne PD, . QuPath: open source software for digital pathology image analysis. Sci Rep 2017;7:16878. 29203879 10.1038/s41598-017-17204-5 PMC5715110

[90] Stringer C , Wang T, Michaelos M, Pachitariu M. Cellpose: a generalist algorithm for cellular segmentation. Nat Methods 2021;18:100–6. 33318659 10.1038/s41592-020-01018-x

[91] Stringer C , Pachitariu M. Cellpose3: one-click image restoration for improved cellular segmentation. Nat Methods 2025;22:592–9. 39939718 10.1038/s41592-025-02595-5 PMC11903308

[92] Achanta R , Shaji A, Smith K, Lucchi A, Fua P, Süsstrunk S. SLIC superpixels compared to state-of-the-art superpixel methods. IEEE Trans Pattern Anal Mach Intell 2012;34:2274–82. 22641706 10.1109/TPAMI.2012.120

[93] Bligh EG , Dyer WJ. A rapid method of total lipid extraction and purification. Can J Biochem Physiol 1959;37:911–17. 13671378 10.1139/o59-099

[94] House RRJ , Soper-Hopper MT, Vincent MP, Ellis AE, Capan CD, Madaj ZB, . A diverse proteome is present and enzymatically active in metabolite extracts. Nat Commun 2024;15:5796. 38987243 10.1038/s41467-024-50128-z PMC11237058

[95] Li Y , Wang C, Chen L. SDAMS: differential abundant/expression analysis for metabolomics, proteomics and single-cell RNA sequencing data. R package version 1.32.0 2026. doi:10.18129/B9.bioc.SDAMS.

[96] Gu Z , Eils R, Schlesner M. Complex heatmaps reveal patterns and correlations in multidimensional genomic data. Bioinformatics 2016;32:2847–9. 27207943 10.1093/bioinformatics/btw313

[97] Panzeri I , Fagnocchi L, Apostle S, Tompkins M, Wolfrum E, Madaj Z, . TRIM28-dependent developmental heterogeneity determines cancer susceptibility through distinct epigenetic states. Nat Cancer 2025;6:385–403. 39856421 10.1038/s43018-024-00900-3 PMC11864977

[98] Berkers CR , Maddocks ODK, Cheung EC, Mor I, Vousden KH. Metabolic regulation by p53 family members. Cell Metab 2013;18:617–33. 23954639 10.1016/j.cmet.2013.06.019 PMC3824073

[99] Cox AR , Chernis N, Masschelin PM, Hartig SM. Immune cells gate white adipose tissue expansion. Endocrinology 2019;160:1645–58. 31107528 10.1210/en.2019-00266 PMC6591013

[100] Miranda AMA , McAllan L, Mazzei G, Andrew I, Davies I, Ertugrul M, . Selective remodelling of the adipose niche in obesity and weight loss. Nature 2025;644:769–79. 40634602 10.1038/s41586-025-09233-2 PMC12367556

[101] Conner SJ , Borges HB, Guarin JR, Gerton TJ, Yui A, Salhany KJ Jr, . Obesity induces temporally regulated alterations in the extracellular matrix that drive breast tumor invasion and metastasis. Cancer Res 2024;84:2761–75. 38900938 10.1158/0008-5472.CAN-23-2526 PMC11908800

[102] Hawes ML , Moody MA, McCauley CR, Huddleston AG, Solanky M, Khosravi DH, . Oncogenic effects of ECM remodeling in obesity and breast cancer. Oncogene 2025;44:3409–21. 40847127 10.1038/s41388-025-03521-x PMC12414810

[103] Tanti J-F , Ceppo F, Jager J, Berthou F. Implication of inflammatory signaling pathways in obesity-induced insulin resistance. Front Endocrinol (Lausanne) 2013;3:181. 23316186 10.3389/fendo.2012.00181 PMC3539134

[104] Heilbronn LK , Campbell LV. Adipose tissue macrophages, low grade inflammation and insulin resistance in human obesity. Curr Pharm Des 2008;14:1225–30. 18473870 10.2174/138161208784246153

[105] Doerner SK , Reis ES, Leung ES, Ko JS, Heaney JD, Berger NA, . High-fat diet-induced complement activation mediates intestinal inflammation and neoplasia, independent of obesity. Mol Cancer Res 2016;14:953–65. 27535705 10.1158/1541-7786.MCR-16-0153 PMC5330314

[106] Wang H , Wan X, Pilch PF, Ellisen LW, Fried SK, Liu L. An AMPK-dependent, non-canonical p53 pathway plays a key role in adipocyte metabolic reprogramming. eLife 2020;9:e63665. 33320092 10.7554/eLife.63665 PMC7758072

[107] Al-Massadi O , Porteiro B, Kuhlow D, Köhler M, Gonzalez-Rellan MJ, Garcia-Lavandeira M, . Pharmacological and genetic manipulation of p53 in brown fat at adult but not embryonic stages regulates thermogenesis and body weight in male mice. Endocrinology 2016;157:2735–49. 27183316 10.1210/en.2016-1209

[108] Dibra D , Mitra A, Newman M, Xia X, Cutrera JJ, Gagea M, . Lack of immunomodulatory interleukin-27 enhances oncogenic properties of mutant p53 in vivo. Clin Cancer Res 2016;22:3876–83. 26979394 10.1158/1078-0432.CCR-15-2052 PMC4970873

[109] Dibra D , Xia X, Mitra A, Cutrera JJ, Lozano G, Li S. Mutant p53 in concert with an interleukin-27 receptor alpha deficiency causes spontaneous liver inflammation, fibrosis, and steatosis in mice. Hepatology 2016;63:1000–12. 26637970 10.1002/hep.28379 PMC4764463

[110] Minamino T , Orimo M, Shimizu I, Kunieda T, Yokoyama M, Ito T, . A crucial role for adipose tissue p53 in the regulation of insulin resistance. Nat Med 2009;15:1082–7. 19718037 10.1038/nm.2014

[111] Liu Y , Su Z, Tavana O, Gu W. Understanding the complexity of p53 in a new era of tumor suppression. Cancer Cell 2024;42:946–67. 38729160 10.1016/j.ccell.2024.04.009 PMC11190820

[112] Liu Y , Gu W. The complexity of p53-mediated metabolic regulation in tumor suppression. Semin Cancer Biol 2022;85:4–32. 33785447 10.1016/j.semcancer.2021.03.010 PMC8473587

[113] Wang P-Y , Ma W, Park JY, Celi FS, Arena R, Choi JW, . Increased oxidative metabolism in the Li–Fraumeni syndrome. N Engl J Med 2013;368:1027–32. 23484829 10.1056/NEJMoa1214091 PMC4123210

[114] Li T , Kon N, Jiang L, Tan M, Ludwig T, Zhao Y, . Tumor suppression in the absence of p53-mediated cell-cycle arrest, apoptosis, and senescence. Cell 2012;149:1269–83. 22682249 10.1016/j.cell.2012.04.026 PMC3688046

[115] Wang P-Y , Ma J, Li J, Starost MF, Wolfgang MJ, Singh K, . Reducing fatty acid oxidation improves cancer-free survival in a mouse model of Li-Fraumeni syndrome. Cancer Prev Res 2021;14:31–40. 10.1158/1940-6207.CAPR-20-0368 PMC7981282 32958587

[116] Wang P-Y , Li J, Walcott FL, Kang JG, Starost MF, Talagala SL, . Inhibiting mitochondrial respiration prevents cancer in a mouse model of Li-Fraumeni syndrome. J Clin Invest 2017;127:132–6. 27869650 10.1172/JCI88668 PMC5199691

[117] Coral DE , Fernandez-Tajes J, Tsereteli N, Pomares-Millan H, Fitipaldi H, Mutie PM, . A phenome-wide comparative analysis of genetic discordance between obesity and type 2 diabetes. Nat Metab 2023;5:237–47. 36703017 10.1038/s42255-022-00731-5 PMC9970876

[118] Khera AV , Chaffin M, Wade KH, Zahid S, Brancale J, Xia R, . Polygenic prediction of weight and obesity trajectories from birth to adulthood. Cell 2019;177:587–96.e9. 31002795 10.1016/j.cell.2019.03.028 PMC6661115

[119] Cirulli ET , Guo L, Leon Swisher C, Shah N, Huang L, Napier LA, . Profound perturbation of the metabolome in obesity is associated with health risk. Cell Metab 2019;29:488–500.e2. 30318341 10.1016/j.cmet.2018.09.022 PMC6370944

[120] Petersen MC , Smith GI, Palacios HH, Farabi SS, Yoshino M, Yoshino J, . Cardiometabolic characteristics of people with metabolically healthy and unhealthy obesity. Cell Metab 2024;36:745–61.e5. 38569471 10.1016/j.cmet.2024.03.002 PMC11025492

[121] Tabara Y , Shoji-Asahina A, Ogawa A, Sato Y. Metabolically healthy obesity and risks of cardiovascular disease and all-cause mortality, a matched cohort study: the Shizuoka study. Int J Obes 2024;48:1164–9. 10.1038/s41366-024-01541-3 38762621

[122] Schulze MB , Stefan N. Metabolically healthy obesity: from epidemiology and mechanisms to clinical implications. Nat Rev Endocrinol 2024;20:633–46. 38937638 10.1038/s41574-024-01008-5

[123] Ślusarz A , Jackson GA, Day JK, Shenouda NS, Bogener JL, Browning JD, . Aggressive prostate cancer is prevented in ERαKO mice and stimulated in ERβKO TRAMP mice. Endocrinology 2012;153:4160–70. 22753646 10.1210/en.2012-1030 PMC3423626

[124] Hillman GG , Wang Y, Kucuk O, Che M, Doerge DR, Yudelev M, . Genistein potentiates inhibition of tumor growth by radiation in a prostate cancer orthotopic model. Mol Cancer Ther 2004;3:1271–9. 15486194

[125] Mentor-Marcel R , Lamartiniere CA, Eltoum IA, Greenberg NM, Elgavish A. Dietary genistein improves survival and reduces expression of osteopontin in the prostate of transgenic mice with prostatic adenocarcinoma (TRAMP). J Nutr 2005;135:989–95. 15867270 10.1093/jn/135.5.989

[126] Liu B , Edgerton S, Yang X, Kim A, Ordonez-Ercan D, Mason T, . Low-dose dietary phytoestrogen abrogates tamoxifen-associated mammary tumor prevention. Cancer Res 2005;65:879–86. 15705886

[127] Singh AV , Franke AA, Blackburn GL, Zhou J-R. Soy phytochemicals prevent orthotopic growth and metastasis of bladder cancer in mice by alterations of cancer cell proliferation and apoptosis and tumor angiogenesis. Cancer Res 2006;66:1851–8. 16452247 10.1158/0008-5472.CAN-05-1332 PMC2683370

[128] Liu F , Peng Y, Qiao Y, Wang P, Si C, Wang X, . Association of urinary phytoestrogens with hormone-related cancers and cancer biomarkers: NHANES 1999–2010. Br J Nutr 2023;130:750–64. 36474419 10.1017/S0007114522003877

[129] Hedelin M , Löf M, Andersson TM-L, Adlercreutz H, Weiderpass E. Dietary phytoestrogens and the risk of ovarian cancer in the women’s lifestyle and health cohort study. Cancer Epidemiol Biomarkers Prev 2011;20:308–17. 21098648 10.1158/1055-9965.EPI-10-0752

[130] Newbold RR , Banks EP, Bullock B, Jefferson WN. Uterine adenocarcinoma in mice treated neonatally with genistein. Cancer Res 2001;61:4325–8. 11389053

[131] Schneider K , Zelley K, Nichols KE, Levine AS, Garber J. Li-Fraumeni syndrome. In: GeneReviews®[Internet]. Seattle (WA): University of Washington; 2025. 20301488

[132] Achatz MI , Villani A, Bertuch AA, Bougeard G, Chang VY, Doria AS, . Update on cancer screening recommendations for individuals with Li–Fraumeni syndrome. Clin Cancer Res 2025;31:1831–40. 40072304 10.1158/1078-0432.CCR-24-3301

[133] Chan CS , Sun Y, Ke H, Zhao Y, Belete M, Zhang C, . Genetic and stochastic influences upon tumor formation and tumor types in Li-Fraumeni mouse models. Life Sci Alliance 2021;4:e202000952. 33376133 10.26508/lsa.202000952 PMC7772779

[134] Donehower LA , Harvey M, Vogel H, McArthur MJ, Montgomery CA Jr, Park SH, . Effects of genetic background on tumorigenesis in p53-deficient mice. Mol Carcinog 1995;14:16–22. 7546219 10.1002/mc.2940140105

[135] Kuperwasser C , Hurlbut GD, Kittrell FS, Dickinson ES, Laucirica R, Medina D, . Development of spontaneous mammary tumors in BALB/c p53 heterozygous mice: a model for Li-Fraumeni syndrome. Am J Pathol 2000;157:2151–9. 11106587 10.1016/S0002-9440(10)64853-5 PMC1885755

[136] Khaddour K , Gomez-Perez SL, Jain N, Patel JD, Boumber Y. Obesity, sarcopenia, and outcomes in non-small cell lung cancer patients treated with immune checkpoint inhibitors and tyrosine kinase inhibitors. Front Oncol 2020;10:576314. 33194687 10.3389/fonc.2020.576314 PMC7607047

[137] Zhang X , Liu Y, Shao H, Zheng X. Obesity paradox in lung cancer prognosis: evolving biological insights and clinical implications. J Thorac Oncol 2017;12:1478–88. 28757418 10.1016/j.jtho.2017.07.022

[138] Assumpção JAF , Pasquarelli-do-Nascimento G, Duarte MSV, Bonamino MH, Magalhães KG. The ambiguous role of obesity in oncology by promoting cancer but boosting antitumor immunotherapy. J Biomed Sci 2022;29:12. 35164764 10.1186/s12929-022-00796-0 PMC8842976

