# Supplementary material for: Chronic High-Fat Diet Does Not Alter Overall Cancer Incidence in Trp53R270H/+ Mice
Source: Cancer Res Commun. 2026 Jun 8;6(6):1336–50. doi: 10.1158/2767-9764.CRC-25-0280 (PMC13244378; doi:10.1158/2767-9764.CRC-25-0280)
Supplement: Supplementary Figure 1 — Longitudinal body and fat mass measurements, adipose and tissue immune staining, lymphocyte gating strategy, and genotype/diet comparisons across female and male mice under chow or high-fat diet conditions. [file crc-25-0280_supplementary_figure_1_suppsf1.pdf]

Suppl.Fig.1

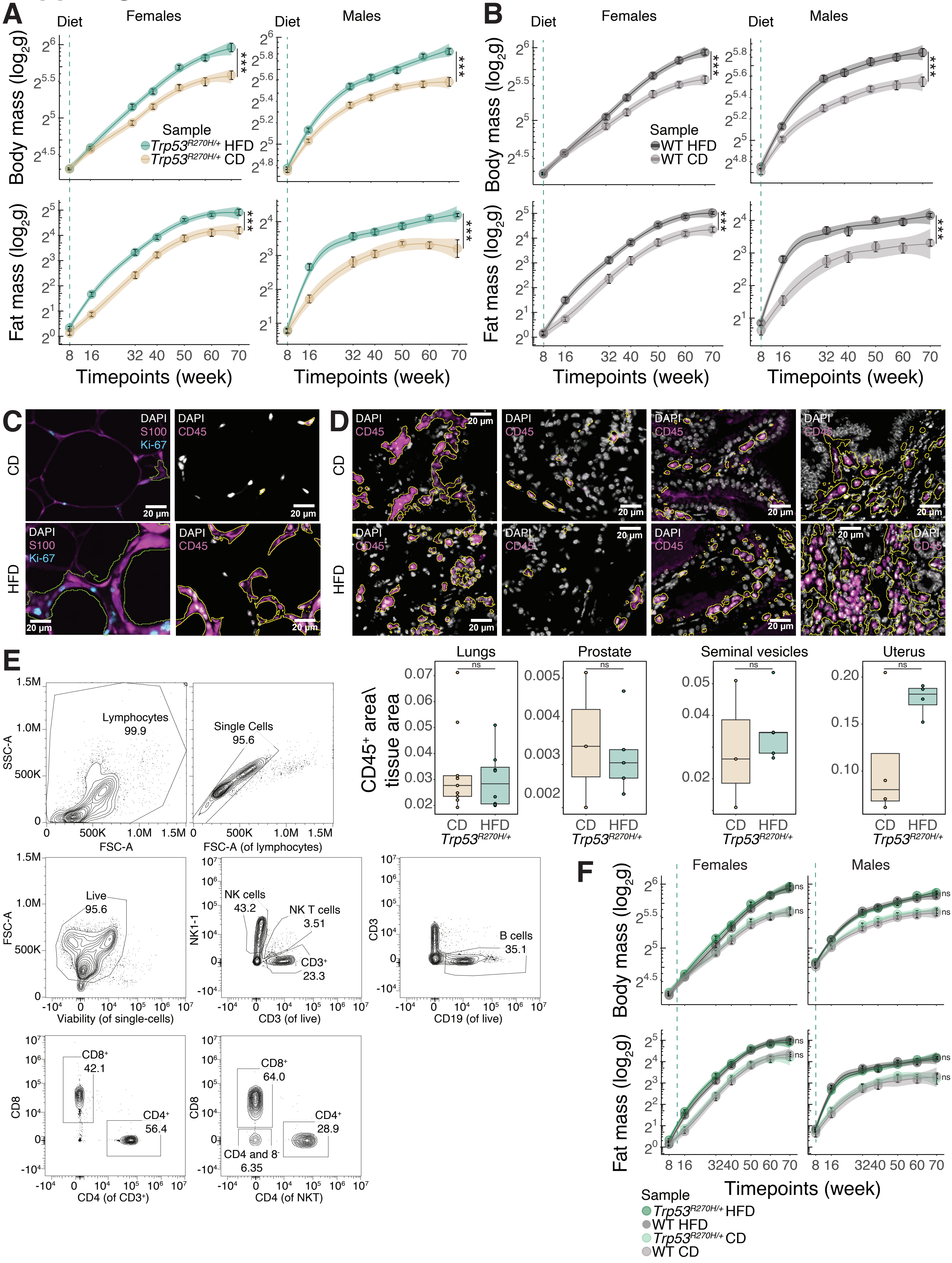
A
Diet
Females
Males
Body mass (log2g)
Fat mass (log2g)
Sample
Trp53R270H/+ HFD
Trp53R270H/+ CD
Timepoints (week)
B
WT HFD
WT CD
C
DAPI
S100
Ki-67
CD45
CD
HFD
20 µm
D
E
Lymphocytes
99.9
Single Cells
95.6
SSC-A
FSC-A
FSC-A (of lymphocytes)
Live
95.6
Viability (of single-cells)
NK cells
43.2
NK T cells
3.51
CD3+
23.3
NK1-1
CD3 (of live)
B cells
35.1
CD3
CD19 (of live)
CD8+
42.1
CD4+
56.4
CD8
CD4 (of CD3+)
CD8+
64.0
CD4+
28.9
CD4 and 8-
6.35
CD4 (of NKT)
Lungs
Prostate
Seminal vesicles
Uterus
CD45+ area\ tissue area
ns
CD
HFD
Trp53R270H/+
F
Sample
Trp53R270H/+ HFD
WT HFD
Trp53R270H/+ CD
WT CD

Supplementary Figure 1. A cohort to study chronic effects of obesity on p53-dependent cancers across females and males. A) Scatter plots and smoothed conditional means (95% confidence interval, “loess” method) for body (top) and fat (bottom) mass in $Trp53^{R270H/+}$ female (left) and male (right) mice under chow (CD, beige) or high-fat (HFD, green) diet. Data reported as $\log_2$ scale. N=101 $Trp53^{R270H/+}$ female (60 HFD-fed and 41 CD-fed) and 94 male (56 HFD-fed and 38 CD-fed) mice. Significant differences were observed in CD vs HFD body and fat mass trajectories with Generalized Additive Models (GAMs) with random effects (Likelihood Ratio Test): p=0 for body and fat mass in females, p=0.0000118 for body and p=0 for fat mass in males. **B)** Scatter plots and smoothed conditional means (95% confidence interval, “loess” method) for body (top) and fat (bottom) mass in wild-type (WT) females (left) and males (right) under CD (beige) or HFD (green). Data reported as $\log_2$ scale. N=70 WT female (40 HFD-fed and 30 CD-fed) and 53 male (29 HFD-fed and 24 CD-fed). mice. Significant differences were observed in CD vs HFD body and fat mass trajectories with GAMs with random effects (Likelihood Ratio Test): p=0 for body and fat mass in females, and p=0.0000003 for body and p=0 for fat mass in males. **C)** Representative examples of S100 and Ki-67 (left), and CD45 (right)-stained epididymal white adipose tissue from CD (top) and HFD (bottom)-fed $Trp53^{R270H/+}$ mice at 70 weeks of age (N=1 male mouse). *Left* – detection of S100 and Ki67-positive areas outlined in Chartreuse Green. *Right* - detection of CD45-positive areas outlined in yellow. Scale bar: 20 µm. **D)** Representative examples of CD45-stained tissues from CD (top) and HFD (bottom)-fed $Trp53^{R270H/+}$ mice at 70 weeks of age (N=1 male mouse) and relative quantifications below (N=9 CD- and N=8 HFD-fed mice for lungs; N=3 CD- and N=5 HFD-fed mice for prostate and seminal vesicles; N=5 CD- and N=4 HFD-fed mice for uterus). Scale bar: 20 µm. No significant differences (ns) were observed for $CD45^+$ area CD vs HFD comparison with Wilcoxon rank rum exact test: W=39, p=0.8148 (lungs); W=9, p=0.7857 (prostate); W=4, p=0.3929 (seminal vesicles); W=4, p=0.3429 (uterus). **E)** Representative FACS gating strategy for lymphocyte identification, single-cell selection, viability, and subset definition from a $Trp53^{R270H/+}$ animal. **F)** Scatter plots and smoothed conditional means (95% confidence interval, "loess" method) for body (top) and fat (bottom) mass in $Trp53^{R270H/+}$ (green shades) and WT (grey shades) female (left) and male (right) mice under CD or HFD. Data reported as $\log_2$ scale. N=101 $Trp53^{R270H/+}$ female (60 HFD-fed and 41 CD-fed), 94 $Trp53^{R270H/+}$ male (56 HFD-fed and 38 CD-fed), 70 WT female (40 HFD-fed and 30 CD-fed), and 53 WT male (29 HFD-fed and 24 CD-fed) mice. No significant differences (ns) were observed between $Trp53^{R270H/+}$ and WT animals within either diet condition with GAMs with random effects (Likelihood Ratio Test).
