# Supplementary material for: Chronic High-Fat Diet Does Not Alter Overall Cancer Incidence in Trp53R270H/+ Mice
Source: Cancer Res Commun. 2026 Jun 8;6(6):1336–50. doi: 10.1158/2767-9764.CRC-25-0280 (PMC13244378; doi:10.1158/2767-9764.CRC-25-0280)
Supplement: Supplementary Figure 2 — Survival analyses, tumor burden distributions, tumor-type prevalence, and tumor-specific survival comparisons in female and male Trp53R270H/+ mice under chow or high-fat diet conditions, including pre- and post-COVID cohorts. [file crc-25-0280_supplementary_figure_2_suppsf2.pdf]

Suppl.Fig.2

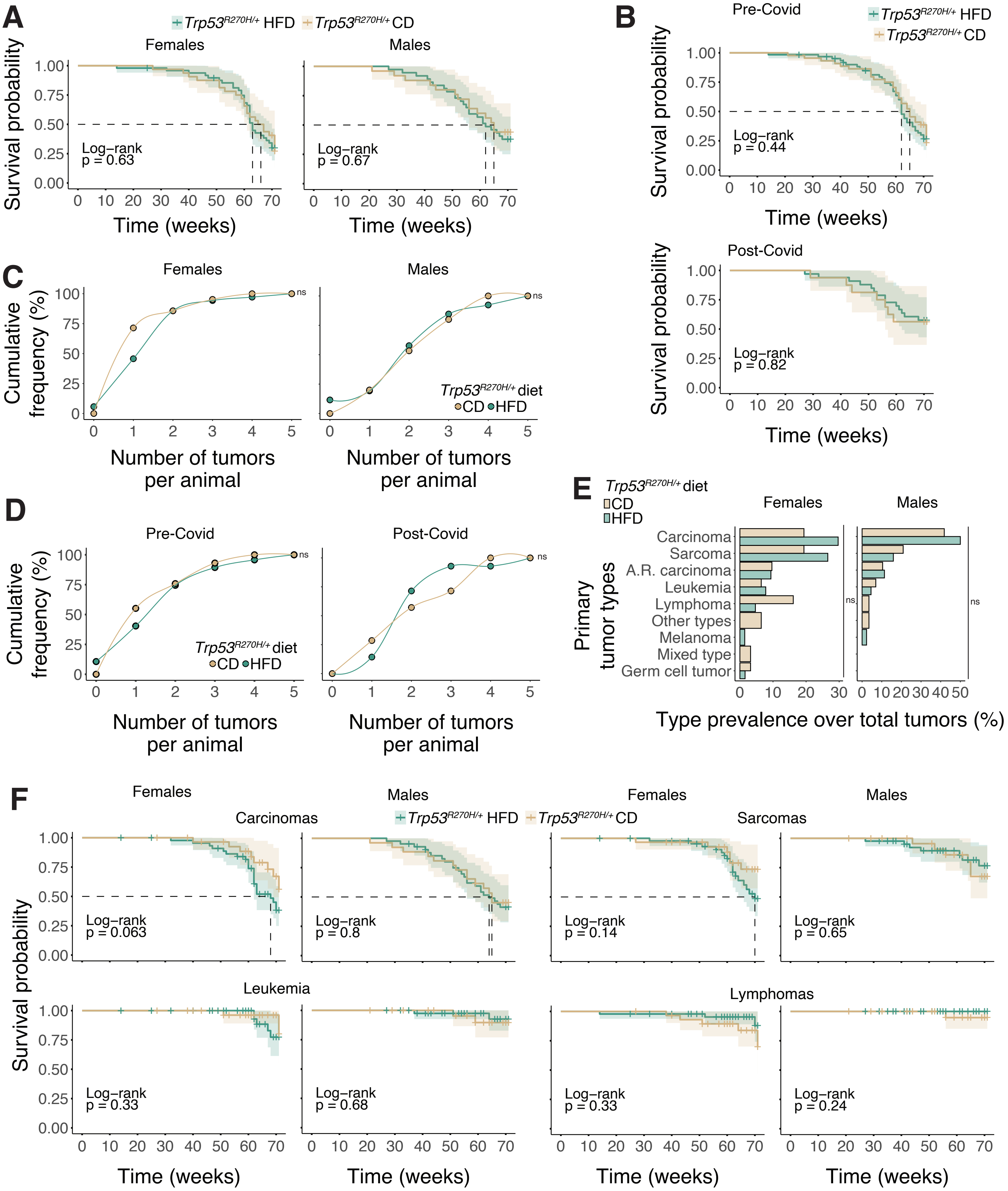
A
Trp53R270H/+ HFD
Trp53R270H/+ CD
Females
Males
Survival probability
Log−rank p = 0.63
Log−rank p = 0.67
Time (weeks)
B
Pre-Covid
Post-Covid
Log−rank p = 0.44
Log−rank p = 0.82
C
Cumulative frequency (%)
Number of tumors per animal
Trp53R270H/+ diet
CD
HFD
ns
D
E
Primary tumor types
Carcinoma
Sarcoma
A.R. carcinoma
Leukemia
Lymphoma
Other types
Melanoma
Mixed type
Germ cell tumor
Type prevalence over total tumors (%)
F
Carcinomas
Sarcomas
Leukemia
Lymphomas
Log−rank p = 0.063
Log−rank p = 0.8
Log−rank p = 0.14
Log−rank p = 0.65
Log−rank p = 0.33
Log−rank p = 0.68
Log−rank p = 0.33
Log−rank p = 0.24

Supplementary Figure 2. HFD does not alter survival nor burden in *Trp53*$^{R270H/+}$ female or male mice. A) Kaplan-Meier survival probability by diet for *Trp53*$^{R270H/+}$ females (left) or males (right). No significant differences were observed according to a Log-rank test: p=0.64 and p=0.67, respectively. N=81 females (49 high-fat diet -HFD- vs 32 chow diet -CD-) and 74 males (46 HFD vs 28 CD). **B)** Kaplan-Meier survival probability by diet for *Trp53*$^{R270H/+}$ mice in the pre-Covid (left) or post-Covid (right) cohorts. No significant differences were observed according to a Log-rank test: p=0.44 and p=0.82, respectively. N=106 pre-Covid (62 HFD vs 44 CD) and 49 post-Covid (33 HFD vs 16 CD). **C)** Cumulative distribution of tumor burden (number of tumors per mouse) in *Trp53*$^{R270H/+}$ females (left) or males (right) fed with CD or HFD. No significant differences (ns) were observed according to a two-sided, two-sample Kolmogorov-Smirnov test for either females (asymptotic; D = 0.037895, p-value = 1) or males (exact; D = 0.039722, p-value = 0.9469). N=56 females (35 HFD vs 21 CD) and 41 males (26 HFD vs 15 CD). **D)** Cumulative distribution of tumor burden (number of tumors per mouse) in *Trp53*$^{R270H/+}$ pre-Covid (left) or post-Covid (right) fed with CD or HFD. No significant differences (ns) were observed according to a two-sided, two-sample Kolmogorov-Smirnov test for either pre-Covid (asymptotic; D = 0.025907, p-value = 1) or post-Covid (exact; D = 0.041538, p-value = 0.9889). N=106 pre-Covid (62 HFD vs 44 CD) and 49 post-Covid (33 HFD vs 16 CD). **E)** Prevalence of each tumor type over the total number of tumors in CD or HFD-fed female or male *Trp53*$^{R270H/+}$ mice. N=95 tumors in females (64 in HFD vs 31 in CD) and 73 in males (44 in HFD vs 29 in CD). No differences (ns) were detected using Poisson regression with log offset for time until euthanasia. A.R. = “age-related”. **F)** Kaplan-Meier survival probability for female and male *Trp53*$^{R270H/+}$ animals under chow (CD, beige) or high-fat (HFD, green) diet for the different tumor types. No significant differences were observed with a Log-rank test for carcinomas (p=0.063 for females, 0.8 for males), sarcomas (p=0.14 for fermales, 0.65 for males), for leukemia (p=0.33 for females, 0.68 for males), and for lymphomas (p=0.33 for females, 0.24 for males). N=81 females (49 high-fat diet -HFD- vs 32 chow diet -CD-) and 74 males (46 HFD vs 28 CD).
