# Supplementary material for: Chronic High-Fat Diet Does Not Alter Overall Cancer Incidence in Trp53R270H/+ Mice
Source: Cancer Res Commun. 2026 Jun 8;6(6):1336–50. doi: 10.1158/2767-9764.CRC-25-0280 (PMC13244378; doi:10.1158/2767-9764.CRC-25-0280)
Supplement: Supplementary Figure 3 — Organ and tumor-type distributions, tumor malignancy frequencies, TRP53 staining analyses, and mutant-to-wild-type Trp53 allele ratios in tumors and matched normal tissues from female and male mice under chow or high-fat diet conditions. [file crc-25-0280_supplementary_figure_3_suppsf3.pdf]

Suppl.Fig.3

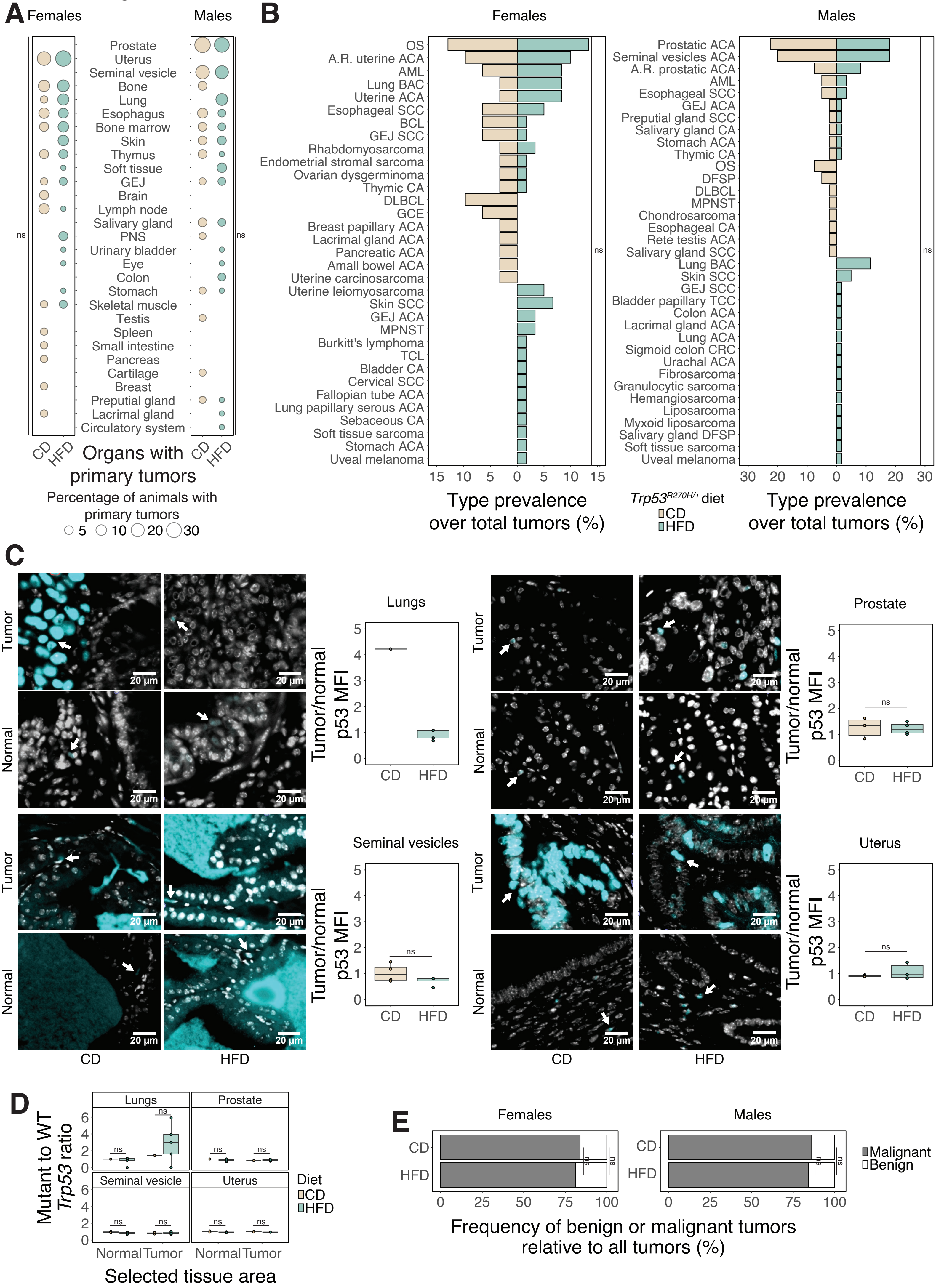
A Females
Males
Prostate
Uterus
Seminal vesicle
Bone
Lung
Esophagus
Bone marrow
Skin
Thymus
Soft tissue
GEJ
Brain
Lymph node
Salivary gland
PNS
Urinary bladder
Eye
Colon
Stomach
Skeletal muscle
Testis
Spleen
Small intestine
Pancreas
Cartilage
Breast
Preputial gland
Lacrimal gland
Circulatory system
ns
CD
HFD
Organs with primary tumors
Percentage of animals with primary tumors
5
10
20
30
B
Females
OS
A.R. uterine ACA
AML
Lung BAC
Uterine ACA
Esophageal SCC
BCL
GEJ SCC
Rhabdomyosarcoma
Endometrial stromal sarcoma
Ovarian dysgerminoma
Thymic CA
DLBCL
GCE
Breast papillary ACA
Lacrimal gland ACA
Pancreatic ACA
Amall bowel ACA
Uterine carcinosarcoma
Uterine leiomyosarcoma
Skin SCC
GEJ ACA
MPNST
Burkitt's lymphoma
TCL
Bladder CA
Cervical SCC
Fallopian tube ACA
Lung papillary serous ACA
Sebaceous CA
Soft tissue sarcoma
Stomach ACA
Uveal melanoma
15 10 5 0 5 10 15
Type prevalence over total tumors (%)
Males
Prostatic ACA
Seminal vesicles ACA
A.R. prostatic ACA
AML
Esophageal SCC
GEJ ACA
Preputial gland SCC
Salivary gland CA
Stomach ACA
Thymic CA
OS
DFSP
DLBCL
MPNST
Chondrosarcoma
Esophageal CA
Rete testis ACA
Salivary gland SCC
Lung BAC
Skin SCC
GEJ SCC
Bladder papillary TCC
Colon ACA
Lacrimal gland ACA
Lung ACA
Sigmoid colon CRC
Urachal ACA
Fibrosarcoma
Granulocytic sarcoma
Hemangiosarcoma
Liposarcoma
Myxoid liposarcoma
Salivary gland DFSP
Soft tissue sarcoma
Uveal melanoma
30 20 10 0 10 20 30
Type prevalence over total tumors (%)
Trp53R270H/+ diet
CD
HFD
C
Tumor
Normal
20 µm
Lungs
Seminal vesicles
Prostate
Uterus
Tumor/normal p53 MFI
0 1 2 3 4 5
CD
HFD
D
Lungs
Prostate
Seminal vesicle
Uterus
Mutant to WT Trp53 ratio
0 2 4 6
Normal
Tumor
Selected tissue area
Diet
CD
HFD
E
Females
Males
Malignant
Benign
0 25 50 75 100
Frequency of benign or malignant tumors relative to all tumors (%)

Supplementary Figure 3. HFD does not alter tumor spectrum nor associated survival in *Trp53*$^{R270H/+}$ female or male mice. A) Percentage of *Trp53*$^{R270H/+}$ female and male mice with primary tumors targeting the different organs. N=54 females (33 HFD vs 21 CD) and 38 males (23 HFD vs 15 CD). No differences (ns) were detected using Poisson regression with log offset for time until euthanasia. **B)** Spectrum of primary tumor types in *Trp53*$^{R270H/+}$ mice. Bar plots show the prevalence of each tumor type relative to the total number of tumors in females (left) and males (right) under chow diet (CD, beige) or high-fat diet (HFD, green). Tumors are grouped by organ site and histological subtype. No differences (ns) were detected using Poisson regression with log offset for time until euthanasia. A.R. = "age-related". **C)** Representative examples of TRP53-stained tissues from tumor and contralateral normal tissues from CD (left) and HFD (right)-fed *Trp53*$^{R270H/+}$ mice (N=1) and relative quantifications (N=1 CD and 5 HFD for lung; N=3 CD and N=4 HFD for prostate; N=4 CD and 4 HFD for seminal vesicles; N=3 CD and 3 HFD for uterus). p53 in cyan, DAPI in white. Arrows mark examples of p53^{+} areas. Scale bar: 20 µm. No significant differences (ns) were observed for p53 tumor/normal MFI ratio in CD vs HFD comparison with Wilcoxon rank sum test with continuity correction for prostate (W=20, p=0.6488), seminal vesicles (W=40, p=0.4282), and uterus (W=12, p=0.3734). **D)** Mutant to WT/ *Trp53*^{R270H} allele ratio in normal and tumor tissues from CD or HFD-fed *Trp53*$^{R270H/+}$ animals (N=1 CD and N=5 HFD for lungs; N=2 CD and 2 HFD for uterus; N=1 CD and 4 HFD for prostate; N=3 CD vs N=4 for seminal vesicles). No significant differences (ns) were observed for the ratio CD vs HFD comparison across all tissues and conditions according to a Wilcoxon rank sum test (W=2, p=1 for normal and W=1, p=0.6667 for tumor in lungs; W=3, p=0.8 for normal and W=1, p=0.8 for tumor in prostate; W=8, p=0.6286 for normal and W=5, p=0.8571 for tumor in seminal vesicles; W=3, p=0.6667 for normal and tumor in uterus). **E)** Frequency of benign or malignant tumors relative to all in *Trp53*$^{R270H/+}$ female and male animals fed with CD or HFD. N=168 malignant and benign tumors (N=31 tumors found in *Trp53*$^{R270H/+}$ females and in 29 males under CD; N=64 tumors found in *Trp53*$^{R270H/+}$ females and 44 in males under HFD). No significant difference (ns) was found in either females or males with a Fisher's exact test for count data: p=1, 95% CI=0.34544445-4.8140587, OR=1.197723 for females; p=1, 95% CI=0.265916-6.090796, OR=1.179755 for males.
