# Supplementary material for: Chronic High-Fat Diet Does Not Alter Overall Cancer Incidence in Trp53R270H/+ Mice
Source: Cancer Res Commun. 2026 Jun 8;6(6):1336–50. doi: 10.1158/2767-9764.CRC-25-0280 (PMC13244378; doi:10.1158/2767-9764.CRC-25-0280)
Supplement: Supplementary Figure 4 — Representative histological images of diverse tumor types identified in the study, including various carcinomas, sarcomas, and lymphomas, with specific examples of metastatic lesions in the lungs and liver across different genotype and diet cohorts. [file crc-25-0280_supplementary_figure_4_suppsf4.pdf]

Suppl.Fig.4

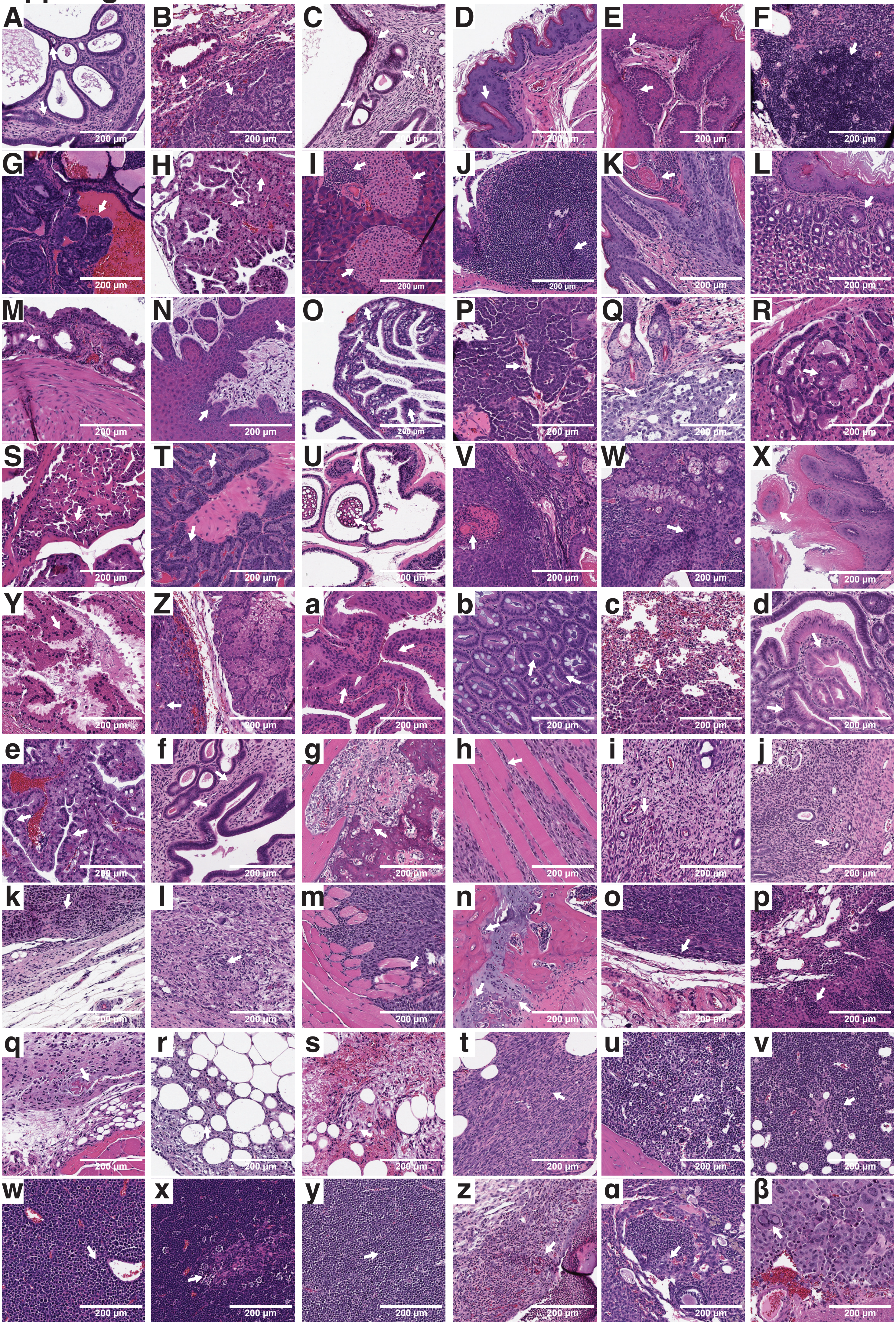
A
B
C
D
E
F
G
H
I
J
K
L
M
N
O
P
Q
R
S
T
U
V
W
X
Y
Z
a
b
c
d
e
f
g
h
i
j
k
l
m
n
o
p
q
r
s
t
u
v
w
x
y
z
α
β
200 µm

Supplementary Figure 4. Representative H&E images for the tumor types observed in the *Trp53*$^{R270H/+}$ cohort. Scale bars: 200 µm. **A)** Age-related uterine carcinoma: arrows mark crowded, back-to-back glands with epithelial stratification and cytologic atypia and cystic dilatation, with minimal stromal reaction. **B)** Lung bronchoalveolar carcinoma: arrows mark the tumor–normal lung interface where atypical pneumocyte-like cells are present with lepidic spread along alveolar septa. **C)** Uterine carcinoma: arrows highlight infiltrative, angulated glands with stromal desmoplasia extending into the myometrium. **D)** Esophageal squamous cell carcinoma: arrow marks an infiltrative squamous nest with keratin-pearl formation extending into the stroma. **E)** Gastro-esophageal junction squamous cell carcinoma: arrows highlight invasive squamous nests breaching the mucosa with accompanying stromal response. **F)** Thymic carcinoma: arrow marks mixed tumor populations comprised of lymphoid and epithelial elements with predominance of carcinoma morphology. **G)** Breast papillary adenocarcinoma: arrow marks papillary epithelial frond with fibrovascular core projection. **H)** Lacrimal gland adenocarcinoma: arrow highlights duct-forming malignant epithelium with cribriform lumina and cytologic atypia. **I)** Islet hypertrophy with inflammation: arrows indicate enlarged endocrine islets and dense peri-islet lymphocytic collection. **J)** Small bowel MALToma: arrow highlights a dense, monotonous infiltrated of uniform lymphoid cells replacing normal mucosal architecture in the proximal caecum (one focus of several lymphoepithelial lesions). **K)** Skin squamous cell carcinoma: arrow denotes invasive keratinizing nest (keratin pearl). **L)** Gastro-esophageal junction adenocarcinoma: arrow highlights infiltrating atypical glands beneath squamous mucosa. **M)** Bladder carcinoma: arrow highlights infiltrating atypical urothelial nests adjacent to muscularis propria. **N)** Cervical squamous cell carcinoma: arrows indicate invasive keratinizing squamous nest within desmoplastic stroma. **O)** Fallopian tube hyperplasia with dysplastic features: arrows mark papillary epithelial frond with fibrovascular core projecting into the lumen. **P)** Lung papillary serous adenocarcinoma: arrow highlights papillary tumor with fibrovascular core lined by atypical tumor cells. **Q)** Sebaceous carcinoma: arrows highlight malignant lobule with foamy/vacuolated cytoplasm consistent with sebaceous differentiation. **R)** Stomach adenocarcinoma: arrow highlights crowded, angulated glands with cribriform high-grade dysplasia. **S)** Prostatic adenocarcinoma: arrow highlights crowded small acini infiltrating fibromuscular stroma. **T)** Seminal vesicles adenocarcinoma: arrows indicate complex cribriform gland with eosinophilic intraluminal secretion. **U)** Age-related prostatic adenocarcinoma: arrows highlight intraductal cribriform growth within a dilated gland and retained prostatic secretions. **V)** Preputial gland squamous cell carcinoma: arrow denotes keratinizing squamous nest (keratin pearl) within desmoplastic stroma. **W)** Salivary gland carcinoma: arrow highlights basaloid nests with solid and cribriform/microcystic pattern invading stroma. **X)** Esophageal squamous cell carcinoma: arrow denotes exophytic keratinizing invasive squamous nest (keratin pearl). **Y)** Rete testis adenocarcinoma: arrow highlights atypical and complex papillary/cribriform gland with invading stroma. **Z)** Salivary gland squamous cell carcinoma: arrow highlights invasive keratinizing squamous nests on the left; benign salivary acini on the right. **a)** Bladder papillary transitional cell carcinoma: arrows mark papillary frond with fibrovascular core and dyshesive atypical urothelium.

b) Colon single gland neoplasia: Arrow highlights solitary gland with high-grade dysplasia**c)** Lung adenocarcinoma: arrow highlights solid, acinar, invasive tumor nests replacing alveolar septa architecture. **d)** Sigmoid colon colorectal carcinoma: arrows highlight polypoid lesion measuring ~1.3 mm with complex cribriform gland with pseudostratified columnar epithelium and cytologic atypia. **e)** Urachal adenocarcinoma: arrows mark complex papillary/cribriform glands with enteric-type mucinous epithelium. **f)** Uterine carcinosarcoma: arrows indicate malignant epithelium and sarcomatous stroma. **g)** Osteosarcoma: sarcomatous proliferation (right), with arrow marking the interface with lace-like osteoid deposition and normal bone marrow elements. **h)** Rhabdomyosarcoma: Arrow marks strap-like rhabdomyoblasts infiltrating skeletal muscle. **i)** Endometrial stromal sarcoma: arrow highlights perivascular whorls of monotonous stromal cells. **j)** Uterine leiomyosarcoma: arrow marks atypical intersecting fascicles of spindle cells with pleomorphic, elongated, blunt-ended, "cigar-shaped" nuclei with hyperchromasia and coarse chromatin. **k)** Malignant peripheral nerve sheet tumor: arrow shows hypercellular intersecting fascicles of atypical spindle cells with wavy nuclei. **l)** Soft tissue sarcoma: arrow highlights interlacing/whorled fascicles of atypical spindle cells with nuclear pleomorphism. **m)** Dermatofibrosarcoma protuberans: arrow marks storiform spindle-cell tumor infiltrating subcutis skeletal muscle. **n)** Chondrosarcoma: arrows indicate atypical chondrocytes within lacunae in hyaline chondroid matrix. **o)** Fibrosarcoma: arrow highlights interlacing spindle-cell fascicles invading collagenous stroma. **p)** Granulocytic sarcoma: Arrow highlights perivascular sheets of monotonous myeloid blasts with fine chromatin and scant cytoplasm. **q)** Hemangiosarcoma: arrow marks irregular vasoformative channel lined by atypical endothelium with associated red blood cells. **r)** Liposarcoma: arrow points at lipoblast/atypical stromal cell within a fibrous septum with hyperchromatic nucleus indented by cytoplasmic lipid vacuoles among variably sized adipocytes. **s)** Myxoid liposarcoma: arrow highlights delicate arborizing capillaries in myxoid matrix with scattered lipoblasts. **t)** Salivary gland dermatofibrosarcoma protuberans: arrow marks storiform spindle-cell proliferation, sweeping/interlacing fascicles of uniform cells. **u)** Acute myeloid leukemia: arrow highlights sheets of myeloblasts with high nuclear-to-cytoplasmic ratio, fine chromatin, and scant cytoplasm replacing marrow. **v)** B-cell lymphoma: arrow points at diffuse sheets of monomorphic atypical lymphoid cells replacing normal architecture. **w)** Diffuse large B-cell lymphoma: arrow marks large atypical lymphoid cells with vesicular chromatin and prominent nucleoli in a diffuse pattern. **x)** Burkitt's lymphoma: arrow highlights tingible-body macrophage creating a "starry-sky" pattern among monomorphic lymphoma cells. **y)** T-cell lymphoma: arrow shows diffuse sheets of atypical T-lymphocytes with irregular nuclear contours and scant cytoplasm. **z)** Uveal melanoma: arrow marks epithelioid/spindle melanoma cells with melanin pigment infiltrating uveal stroma. **α)** Ovarian dysgerminoma: Arrow indicates fibrous septum with lymphocytes bordering nests of polygonal tumor cells with clear cytoplasm. Arrow points at fibrous septum with lymphocytes bordering nests of polygonal tumor cells with clear cytoplasm. **β)** Giant cell ependymoma: arrow highlights a perivascular pseudorosette with adjacent pleomorphic giant cells.
