# Supplementary material for: Chronic High-Fat Diet Does Not Alter Overall Cancer Incidence in Trp53R270H/+ Mice
Source: Cancer Res Commun. 2026 Jun 8;6(6):1336–50. doi: 10.1158/2767-9764.CRC-25-0280 (PMC13244378; doi:10.1158/2767-9764.CRC-25-0280)
Supplement: Supplementary Figure 5 — Comparison of tumor-free survival and overall survival curves between Trp53(R270H/+) and wild-type mice, stratified by sex and diet, along with quantitative analysis of tumor multiplicity and the distribution of primary versus metastatic lesions. [file crc-25-0280_supplementary_figure_5_suppsf5.pdf]

Suppl.Fig.5

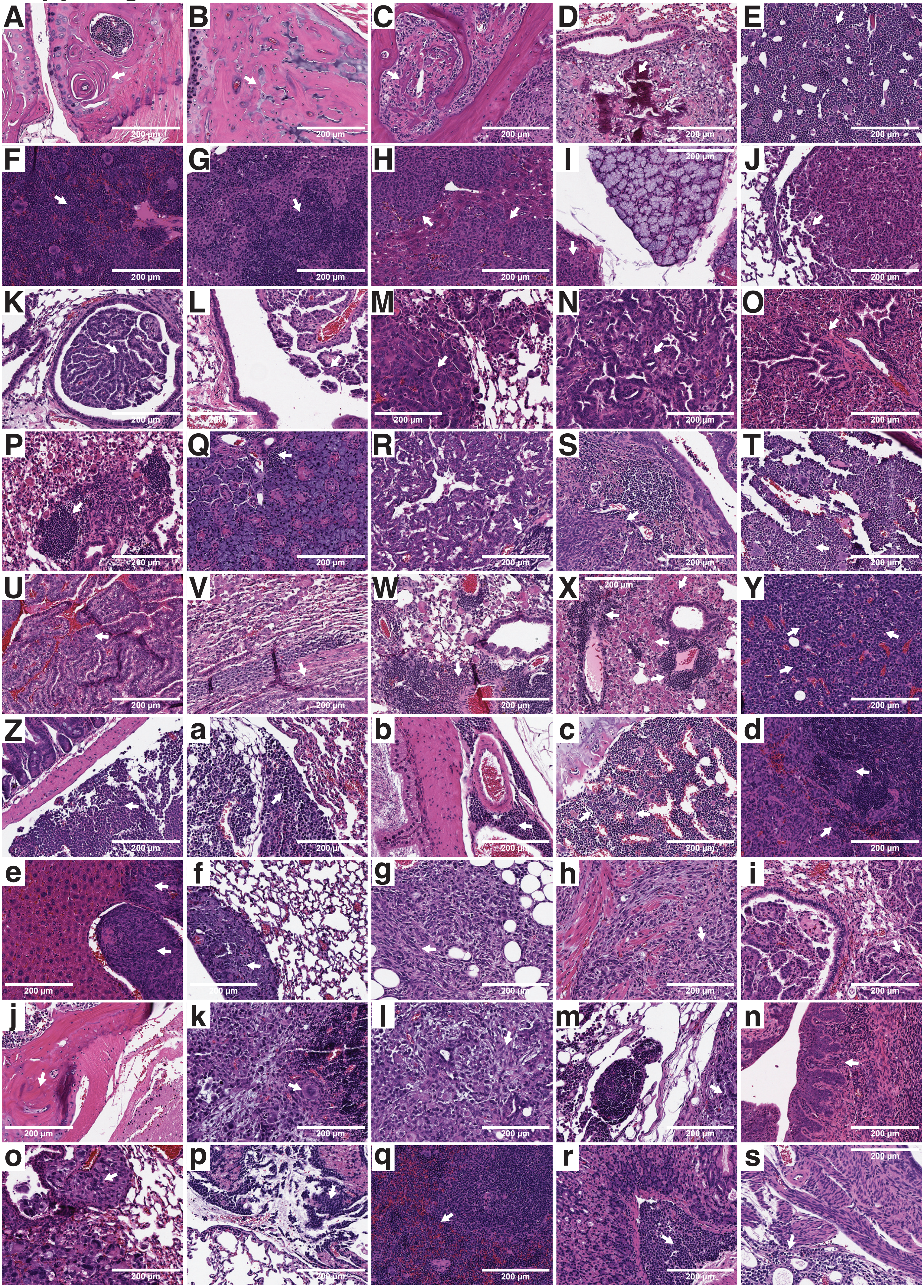

A
B
C
D
E
F
G
H
I
J
K
L
M
N
O
P
Q
R
S
T
U
V
W
X
Y
Z
a
b
c
d
e
f
g
h
i
j
k
l
m
n
o
p
q
r
s
200 µm

Supplementary Figure 5. Representative H&E images for the primary tumors and relative metastasis observed in the *Trp53$^{R270H/+}$* cohort. Scale bars: 200 µm. **(A-D)** Osteosarcoma with solitary lung metastasis. Panels **A–B** show the primary osteosarcoma arising in the long bone, with arrows indicating malignant lace-like osteoid and atypical osteoblastic cells. **C** shows involvement of the spine, with arrows marking irregular osteoid matrix surrounded by pleomorphic tumor cells. **D** depicts the solitary metastatic focus in the lung, where arrows highlight infiltrative malignant cells replacing alveolar architecture. **(E–J)** Acute myeloid leukemia (AML) with multisite myeloid infiltration and a separate bronchoalveolar-type lung tumor. **E** shows bone marrow effaced by crowded immature myeloid cells with loss of megakaryocytes, consistent with AML. **F** shows splenic involvement with expanded red pulp and fused myeloid regions. **G** depicts myeloid tumor deposits within a lymph node. **H** shows classic hepatic involvement with peri–central vein myeloid infiltration. **I** demonstrates myeloid tumor deposits extending into adipose and adjacent submandibular gland tissue. **J** shows a distinct lung lesion with papillary architecture, consistent with a separate BAL-type tumor. **(K–L)** Multifocal bronchoalveolar carcinoma (BAL) of the lung. Panels show characteristic space-occupying bronchoalveolar carcinoma nodules with minimal stromal invasion; arrows indicate papillary and lepidic growth of atypical pneumocyte-like cells along alveolar and bronchiolar surfaces. **(M–T)** Multifocal - both within same lobe and separate lobes - lung adenocarcinoma with aggressive features. Panels **M–O** show three distinct invasive adenocarcinoma foci within the lung. **P** shows an additional lung focus with dense inflammatory and tumor cell infiltrates. **Q** depicts a benign submandibular gland adenoma, **R** shows papillary carcinoma arising within the ovarian tissue, **S** shows endocervix with asymmetric stromal immune response suggestive of invasive tumor, and **T** shows normal rib bone with trilineage maturation. **(U–X)** Lung adenocarcinoma with admixed hepatoid bronchoalveolar carcinoma (BAC) and prominent peri-vascular immune infiltrates. Panel **U** shows the central BAC/BAL component with papillary and lepidic tumor growth; the arrow highlights atypical epithelial cells lining alveolar structures. **V** depicts the tumor margin, where the BAC architecture transitions into adjacent parenchyma. **W** demonstrates the striking peri-vascular immune infiltrate described by the pathologist, which differentiates this case from classic BAL/BAC. **X** shows areas with hepatoid differentiation, with arrows marking clusters of large eosinophilic tumor cells that cause marked immune response. **(Y–b)** High-grade lymphoma with typical lymph-node-based distribution and intermittent serosal spread across thoracic and abdominal organs. Panel **Y** shows thymic involvement with diffuse sheets of monomorphic atypical lymphoid cells replacing the native architecture. **Z** demonstrates serosal and subserosal intestinal infiltration, with the arrow highlighting tumor cells extending along the bowel surface. Panel **a** shows pulmonary involvement with lymphoid aggregates infiltrating alveolar septa. Panel **b** depicts lymphomatous infiltration of the testis and adjacent soft tissues, with the arrow marking serosal/vascular-adjacent clusters of neoplastic lymphoid cells. **(c–f)** Acute myeloid leukemia (AML) with multisite involvement, including liver metastases, vascular dissemination, and a pleural lung focus. Panel **c** shows AML infiltration in bone with sheets of immature myeloid blasts replacing normal marrow spaces. **d** demonstrates splenic involvement with dense myeloid infiltrates and adjacent areas

showing early infarction. **e** shows diffuse hepatic infiltration, with arrows marking peri-vascular and peri-sinusoidal clusters of leukemic blasts. **f** depicts a pleural-based lung focus, with arrows highlighting leukemic cells extending along pleural and subpleural regions. **(g–j)** Multifocal neoplastic involvement including soft tissue sarcoma, cardiac infiltration, a brain-associated bone lesion, and bronchoalveolar-type lung changes. Panel **g** shows a soft tissue sarcoma infiltrating adipose tissue, with the arrow indicating atypical spindle cells within fibrous stroma. **h** depicts tumor infiltration of the heart, with arrows highlighting malignant spindle cells interspersed among myocardial fibers. **i** shows a separate lung primary tumor with bronchoalveolar-type proliferation in the lung, with the arrow marking atypical epithelial nests lining alveolar spaces. **j** shows a meninges lesion with features suggestive of meningioma-associated bone involvement, with the arrow highlighting atypical osteoid and associated spindle-cell proliferation. **(k–o)** Biphasic, poorly differentiated thymic carcinoma with aggressive thoracic and metastatic spread. Panel **k** shows the primary thoracic lesion with biphasic morphology, including sheets of atypical epithelial cells admixed with spindle-cell areas; the arrow highlights a cohesive epithelial nest. **l** depicts tumor infiltration near the spine, with the arrow marking poorly differentiated epithelial clusters extending into paraspinal soft tissues. **m** shows metastatic spread into subcutaneous adipose tissue, with the arrow indicating infiltrating tumor nests between adipocytes. **n** demonstrates involvement of the uterus, with the arrow marking invasive epithelial clusters within the myometrium/serosa. **o** shows metastatic deposits in the lung, with the arrow highlighting tumor infiltration into alveolar/parenchymal structures.
