# Supplementary material for: Chronic High-Fat Diet Does Not Alter Overall Cancer Incidence in Trp53R270H/+ Mice
Source: Cancer Res Commun. 2026 Jun 8;6(6):1336–50. doi: 10.1158/2767-9764.CRC-25-0280 (PMC13244378; doi:10.1158/2767-9764.CRC-25-0280)
Supplement: Supplementary Table 3 — Nucleotide sequences for the forward and reverse primers used for the PCR-based genotyping of the Trp53 alleles in the experimental mouse cohort. [file crc-25-0280_supplementary_table_3_suppst3.docx]

**Supplementary Table 3 – Genotyping primers.**

| Target | Forward primer | Reverse primer |
| --- | --- | --- |
| *Trp53* | ATGCGACTCTCCAGCCTTGGTA | TTGGGCTTAGGGACGTCTCTTATC |
