# Supplementary material for: Chronic High-Fat Diet Does Not Alter Overall Cancer Incidence in Trp53R270H/+ Mice
Source: Cancer Res Commun. 2026 Jun 8;6(6):1336–50. doi: 10.1158/2767-9764.CRC-25-0280 (PMC13244378; doi:10.1158/2767-9764.CRC-25-0280)
Supplement: Supplementary Table 4 — Summary of the thermal cycling parameters, including temperatures and durations for denaturation, annealing, and extension phases, used for the PCR amplification of the Trp53 gene. [file crc-25-0280_supplementary_table_4_suppst4.docx]

**Supplementary Table 4 – Genotyping PCR conditions.**

| Target | Initial denaturation | Denaturation  (x40 cycles) | Annealing  (x40 cycles) | Extension  (x40 cycles) | Final extension | Hold |
| --- | --- | --- | --- | --- | --- | --- |
| *Trp53* | 95 ℃ for 3 minutes | 95 ℃ for 30 seconds | 63 ℃ for 30 seconds | 72 ℃ for 1 minute | 72 ℃ for 5 minutes | 4 ℃ infinite |
