# Supplementary material for: Chronic High-Fat Diet Does Not Alter Overall Cancer Incidence in Trp53R270H/+ Mice
Source: Cancer Res Commun. 2026 Jun 8;6(6):1336–50. doi: 10.1158/2767-9764.CRC-25-0280 (PMC13244378; doi:10.1158/2767-9764.CRC-25-0280)
Supplement: Supplementary Table 5 — Details of the restriction enzyme digestion protocol, including specific incubation temperatures and times for the MslI enzyme and subsequent heat inactivation, used for Trp53 allele discrimination. [file crc-25-0280_supplementary_table_5_suppst5.docx]

**Supplementary Table 5 – Genotyping restriction conditions.**

| Target | Digestion | Enzyme inactivation | Hold |
| --- | --- | --- | --- |
| *Tp53* | 37 ℃ for 30 minutes (MslI) | 80°C for 20 minutes | 4 ℃ infinite |
