# Supplementary material for: Chronic High-Fat Diet Does Not Alter Overall Cancer Incidence in Trp53R270H/+ Mice
Source: Cancer Res Commun. 2026 Jun 8;6(6):1336–50. doi: 10.1158/2767-9764.CRC-25-0280 (PMC13244378; doi:10.1158/2767-9764.CRC-25-0280)
Supplement: Supplementary Table 6 — Sequences for the forward and reverse primers and allele-specific probes used for the quantitative PCR-based discrimination between wild-type and mutant Trp53 alleles. [file crc-25-0280_supplementary_table_6_suppst6.docx]

**Supplementary Table 6 – ddPCR primers and probes.**

| Forward primer | CCTTGTGCTGGTCCTT |
| --- | --- |
| Reverse primer | TACGGCGGTCTCTCC |
| WT Probe | CAGCTTTGAGGTTCGTGTTTGT |
| Mutant Probe | AGCTTTGAGGTTCATGTTTGTGC |
